# The persistent challenge of ischemic stroke burden from high fasting plasma glucose: a global perspective

**DOI:** 10.3389/fendo.2025.1490428

**Published:** 2025-05-06

**Authors:** Zhenhai Sun, Menghe Zhang, Yaoyao Zuo, Wenwen Li, Shudi Li, Yunxiao Zhang, Shouqiang Chen

**Affiliations:** ^1^ Second School of Clinical Medicine, Shandong University of Traditional Chinese Medicine, Jinan, China; ^2^ Department of Cardiology, The Second Affiliated Hospital of Shandong University of Traditional Chinese Medicine, Jinan, China

**Keywords:** global burden, high fasting plasma glucose, ischemic stroke, disability-adjusted life years, mortality

## Abstract

**Background:**

Ischemic stroke is a leading cause of disability and mortality worldwide, with high fasting plasma glucose (HFPG) recognized as a critical modifiable risk factor. This study aimed to evaluate the global disease burden of ischemic stroke attributable to HFPG and predict trends over the next 15 years.

**Methods:**

We utilized the comparative risk assessment method from the Global Burden of Disease (GBD) 2021 study to quantify disease burden in terms of deaths, Disability-Adjusted Life Years (DALYs), and their age-standardized rates. The estimated annual percent changes (EAPCs) were calculated to evaluate temporal trends. Additionally, our analysis included health inequality analysis, decomposition analysis, and predictive analysis employing the Bayesian Age-Period-Cohort model (BAPC).

**Results:**

In 2021, the global deaths and DALYs attributable to ischemic stroke due to HFPG were 659,378 (95% UI: 507,502 to 823,945) and 12,371,434 (95% UI: 9,587,506 to 15,382,662), respectively. Notably, both figures have doubled since 1990. Over the past 30 years, both the age-standardized mortality rate (ASMR) and the age-standardized DALY rate (ASDR) have experienced a significant decline, with EAPC of -0.96 (95% CI: -1.05 to -0.86) and -0.72 (95% CI: -0.81 to -0.62), respectively. High-middle and middle Socio-Demographic Index (SDI) regions represented the primary locations of disease burden, while this burden is gradually shifting towards low SDI regions. Furthermore, the burden was more significant in men than in women and was more pronounced in middle-aged and elderly populations compared to younger individuals. Population growth and aging were the principal factors contributing to the increasing disease burden. Additionally, projections indicate that the disease burden will exhibit a downward trend over the next 15 years.

**Conclusion:**

For over 30 years, while ASMR and ASDR have shown a decline, the deaths and DALYs attributable to ischemic stroke resulting from HFPG continue to rise globally. This trend underscores the persistent public health challenge posed by ischemic stroke associated with HFPG. Future targeted medical strategies should prioritize populations beyond those with High SDI, especially concentrating on middle-aged and elderly individuals and male patients.

## 1 Introduction

Stroke encompasses a range of acute cerebrovascular diseases resulting from either hemorrhage or ischemia. These conditions are characterized by high rates of morbidity, mortality, disability, and recurrence, affecting approximately 13.7 million people worldwide each year and posing a significant threat to global public health (1). Among the various subtypes of stroke, ischemic stroke is particularly significant, accounting for 80% of all stroke cases (2). By 2019, ischemic stroke accounted for 3.29 million fatalities and resulted in 63.48 million Disability-Adjusted Life Years (DALYs) (3). Catastrophic health expenditures during this period totaled an alarming US$964.51 billion. This significant financial burden on healthcare systems represented approximately 0.78% of the world’s GDP (4). Despite advancements in addressing the challenges associated with ischemic stroke, it remains a critical public health issue, imposing substantial financial and healthcare burdens.

Previous studies have demonstrated that excessive blood glucose levels can directly lead to significant health issues such as diabetes or indirectly increase susceptibility to related diseases, including ischemic heart disease and ischemic stroke (5, 6). Research has confirmed that hyperglycemia can independently enlarge the lesion area following ischemic stroke and elevate associated mortality and disability rates (7). Additionally, another study indicated that hyperglycemia may diminish the clinical efficacy of thrombolysis or thrombectomy, which are important treatments for patients with ischemic stroke. This reduction in efficacy may lead to increased patient mortality and worsening of neurological impairment, ultimately resulting in a poorer prognosis (8). However, because hyperglycemia is primarily characterized by elevated blood sugar levels in the early stages and rarely presents obvious clinical symptoms, there is a lack of understanding regarding its potential health impacts. Notably, the Global Burden of Disease (GBD) framework defines high fasting plasma glucose (HFPG) as fasting blood glucose levels exceeding 4.8 - 5.4 mmol/L (theoretical minimum risk exposure level, TMREL) (9). This standard is grounded in continuous risk evidence and encompasses the subclinical population that does not meet the diabetes threshold but experiences a long-term cumulative increase in stroke risk. This definition is instrumental in shaping public health policies and offers a scientific foundation for early intervention and prevention strategies.

Previous studies have primarily concentrated on the impact of HFPG on the overall disease burden or the burden of stroke attributable to various comprehensive risk factors (10, 11). However, few studies have quantified the specific burden of HFPG on ischemic stroke in isolation. While a regional study has reported time trends of HFPG-related ischemic stroke burden in China, a systematic global assessment remains lacking, particularly concerning long-term trends, gender and age heterogeneity, and future predictions (12). We hypothesize that HFPG plays a significant but underrecognized role in the burden of ischemic stroke, particularly in older and male populations. Therefore, to systematically evaluate the impact of HFPG on ischemic stroke burden, we analyzed the global age- and sex-specific disease burden using data from the GBD 2021 study. The analysis also included trends in spatial distribution and temporal distribution. Additionally, we projected the global disease burden over the next 15 years. The findings of this study may provide valuable evidence for policymakers to assess and formulate short- and long-term strategies for managing ischemic stroke linked to HFPG.

## 2 Materials and methods

### 2.1 Data source and definitions

HFPG-attributable ischemic stroke data from 1990 to 2021 were obtained using the Global Health Data Exchange (GHDx) query tool (http://ghdx.healthdata.org/gbd-results-tool). This dataset encompasses various genders, age groups, countries, regions, and socio-demographic index (SDI) classifications. It also records the deaths and DALYs, which combine the years of life lost due to premature mortality and the years lived with disability, as well as the age-standardized mortality rate (ASMR) and age-standardized DALYs rate (ASDR), along with their respective 95% uncertainty intervals (95% UI) (13). As described above, the general approach to estimating disease burden indicators has been elucidated in prior publications (13). The comprehensive index SDI value, calculated by integrating factors such as per capita income, education level, and the total fertility rate of women under 25 years old, provides insight into the social and population development levels of each region (14). According to the SDI value, the world can be categorized into five distinct regions: low, low-middle, middle, high-middle, and high (14).

In this research, the disease burden of ischemic stroke attributable to HFPG was assessed through the Comparative Risk Assessment (CRA) method, based on the GBD 2021 framework. The World Health Organization defines ischemic stroke as a form of neurological impairment caused by reduced or stopped blood flow to brain tissue, often resulting from atherosclerosis or thromboembolism (15). The GBD 2021 defines ischemic stroke in accordance with the 10th revision of the International Classification of Diseases (ICD-10). The relevant diagnostic codes include G45-G46.8, I63-I63.9, I65-I66.9, I67.2-I67.848, and I69.3-I69.4 (11). HFPG is defined within the GBD 2021 as a fasting blood glucose level exceeding the TMREL (4.8–5.4 mmol/L), which is significantly lower than the diabetes diagnostic threshold (≥7.0 mmol/L). This range is derived from a meta-analysis of prospective cohort studies and calculated using the weighted average fasting plasma glucose level per person-year, which correlates with the lowest risk of ischemic stroke. In contrast to clinical guidelines that emphasize individual diagnosis, the GBD framework highlights the impact of any blood glucose level that deviates from TMREL on the population burden of ischemic stroke (16).

### 2.2 HFPG Attribution Burden Estimation Method

To delineate the impact of HFPG on the burden of ischemic stroke, GBD 2021 employs the CRA framework (9, 16). First, the HFPG exposure distribution across various countries, regions, genders, and age groups is estimated using the spatiotemporal Gaussian process regression (ST-GPR) model (16). Then, the relative risk (RR) of HFPG related to ischemic stroke is calculated based on a dose-response meta-analysis of prospective cohort studies, adjusting for potential confounders such as age, gender, and region (16). Finally, the population attributable fraction (PAF)—the proportion of disease burden that could be alleviated if HFPG exposure levels were restricted to the TMREL(4.8–5.4 mmol/L)—is computed, followed by an estimation of the ischemic stroke deaths and DALYs burden attributable to HFPG (16). Importantly, the CRA framework evaluates the contribution of each risk factor independently by calculating the PAF and adjusting for potential confounding variables (16).

### 2.3 Cross-country inequality analysis

This study reveals cross-national health inequalities through the concentration index (CI) and the slope index of inequality (SII). Precisely, the SII is calculated by performing linear regression of death rate (per 100,000 population) or DALY rate (per 100,000 population) against the SDI-related location scales. The SII measures absolute health inequality, where higher values indicate greater disparity. In contrast, the CI, calculated using the Lorenz concentration curve, assesses relative inequality; higher values reflect more inequality. A positive CI denotes that the disease burden is greater in wealthier countries, whereas a negative CI suggests it is higher in poorer countries (17).

### 2.4 Decomposition analysis

The decomposition analysis developed by Das Gupta offers valuable insights into the specific factors contributing to changes in disease burden indicators. Utilizing this analysis, we decompose the variations in DALYs and deaths through three primary factors: population aging, population growth, and epidemiological changes. This approach allows us to understand better the contributions of different factors to the overall changes (18).

### 2.5 Predictive analysis

This study employed the Bayesian Age-Period-Cohort (BAPC) model. Utilizing the R package “BAPC” and integrated nested Laplace approximation (INLA), it predicted gender-specific mortality and DALY rates from 2022 to 2036 (19). The model was based on historical data from 1990 to 2021 in the GBD 2021 database and operated under the following assumptions: (1) Age, period, and cohort effects were smoothed through a second-order random walk (RW2) prior, implying that epidemiological trends, such as population aging, growth patterns, and disease mortality, would continue along their historical trajectories; (2) There would be no significant mutations in socioeconomic development, medical interventions, and risk factors during the prediction period (20).

### 2.6 Statistical analysis

Based on classifications of gender, age, and location, we utilized data on deaths, DALYs, ASMR, and ASDR data, including their corresponding 95% UI, to assess the disease burden. The estimated annual percent changes (EAPC) indicator was introduced to assess disease burden trends from 1990 to 2021. The EAPC is derived from the log-linear regression equation ln(ASR) = α + βX + ϵ , where ASR denotes the age-standardized rate, β represents the regression coefficient, X represents the year. The EAPC finally is calculated as 100 × [exp(β) – 1], accompanied by a 95% confidence interval (95% CI) (21). If the lower bound of the EAPC’s 95% CI is above zero, it indicates an upward trend in ASMR and ASDR; otherwise, the trend is downward (22). All figures and tables were generated using R software version 4.2.3.

## 3 Results

### 3.1 Global ischemic stroke burden attributable to HFPG from 1990 to 2021

Globally, the total number of ischemic stroke deaths due to HFPG increased by 98% between 1990 and 2021, rising from 332,991 (95% UI: 259,792 to 416,391) to 659,378 (95% UI: 507,502 to 823,945) (**Table 1**). The DALYs have also changed like that of deaths. Specifically, the number of DALYs has more than doubled, rising from 6,189,756 (95% UI: 4,834,685 to 7,817,249) in 1990 to 12,371,434 (95% UI: 9,587,506 to 15,382,662) in 2021 (**Table 2**).

**Table 1:** Deaths and ASMR of ischemic stroke attributable to HFPG in 1990 and 2021, and trends over this period.

Deaths	1990		2021		1990–2021
Location	Deaths cases No. (95% UI)	ASMR per 100,000 No. (95% UI)	Deaths cases No. (95% UI)	ASMR per 100,000 No. (95% UI)	EAPC in ASMR No. (95% CI)
Global	332991 (259792,416391)	10.55 (8.18,13.16)	659378 (507502,823945)	8.11 (6.23,10.14)	-0.96 (-1.05,-0.86)
Sex					
Male	149420 (117493,187804)	11.59 (9.08,14.58)	326783 (254090,406557)	9.43 (7.31,11.75)	-0.73 (-0.81,-0.64)
Female	183571 (140508,230874)	9.77 (7.46,12.29)	332595 (251562,421804)	7.07 (5.35,8.96)	-1.2 (-1.31,-1.09)
SDI					
Low SDI	11045 (8141,14940)	7.57 (5.55,10.14)	28790 (21143,37739)	8.26 (6.07,10.75)	0.29 (0.22,0.35)
Low-middle SDI	36623 (28242,47452)	8.61 (6.58,11.13)	112810 (85643,141013)	9.94 (7.54,12.4)	0.48 (0.42,0.54)
Middle SDI	71778 (55496,93035)	10.05 (7.78,13)	208524 (159415,264319)	9.18 (7,11.65)	-0.25 (-0.36,-0.14)
High-middle SDI	118568 (92777,147443)	14.95 (11.56,18.59)	203347 (155682,253067)	10.52 (8.03,13.08)	-1.32 (-1.52,-1.12)
High SDI	94328 (72502,117054)	8.44 (6.45,10.47)	105070 (77622,130058)	4.03 (3.03,4.97)	-2.68 (-2.82,-2.54)
GBD region					
Oceania	149 (109,199)	9.3 (6.93,12.31)	398 (291,536)	8.96 (6.61,11.92)	-0.23 (-0.3,-0.16)
East Asia	66742 (50905,88432)	11.39 (8.63,15.04)	205418 (151603,265997)	10.71 (7.89,13.92)	-0.02 (-0.33,0.29)
Southeast Asia	17577 (13515,22483)	10.12 (7.75,12.9)	57014 (41776,73333)	11.59 (8.5,14.85)	0.53 (0.36,0.71)
Central Asia	3298 (2483,4201)	8.25 (6.21,10.55)	7202 (5537,9086)	11.46 (8.77,14.42)	0.82 (0.59,1.05)
Central Europe	30381 (23817,37596)	23.53 (18.41,29.13)	35081 (27156,43553)	14.3 (11.07,17.76)	-1.93 (-2.08,-1.77)
High-income Asia Pacific	20965 (16226,25850)	12.46 (9.54,15.48)	24172 (17222,30818)	3.39 (2.47,4.25)	-4.56 (-4.71,-4.4)
Western Europe	55453 (42579,69085)	9.03 (6.91,11.25)	38963 (28437,49141)	3.02 (2.23,3.8)	-3.74 (-3.87,-3.62)
Eastern Europe	41670 (32387,53292)	17.19 (13.33,21.98)	49644 (37694,62747)	13.64 (10.36,17.28)	-1.42 (-1.86,-0.98)
Australasia	1477 (1096,1858)	6.71 (4.94,8.53)	1804 (1297,2275)	2.73 (1.98,3.45)	-3.11 (-3.2,-3.01)
Southern Latin America	3414 (2628,4283)	8.31 (6.36,10.38)	4017 (3019,4977)	4.32 (3.25,5.35)	-1.77 (-1.9,-1.64)
High-income North America	18263 (13841,22841)	4.86 (3.68,6.1)	30720 (22515,37819)	4.09 (3.01,5.02)	-1.04 (-1.33,-0.75)
Caribbean	2070 (1606,2570)	9.17 (7.13,11.32)	3985 (2989,5020)	7.28 (5.46,9.18)	-0.69 (-0.79,-0.6)
South Asia	27166 (20042,36425)	7.01 (5.25,9.37)	89851 (68536,117916)	7.74 (5.84,10.12)	0.16 (0.08,0.24)
Central Latin America	4552 (3563,5633)	7.06 (5.5,8.77)	8890 (6826,11239)	3.87 (2.97,4.89)	-2.16 (-2.33,-2)
Andean Latin America	667 (504,850)	3.92 (2.95,4.97)	1775 (1297,2294)	3.23 (2.36,4.17)	-0.76 (-0.88,-0.64)
Tropical Latin America	8949 (6945,11210)	12.99 (9.92,16.39)	14322 (10924,17733)	5.93 (4.52,7.37)	-2.23 (-2.36,-2.09)
North Africa and Middle East	19505 (14858,25166)	16.41 (12.64,21.15)	57196 (43179,71670)	16.83 (12.74,21.13)	0.13 (0.1,0.16)
Southern Sub-Saharan Africa	1301 (952,1677)	6.47 (4.7,8.36)	4349 (3352,5450)	10.53 (8.04,13.33)	1.9 (1.39,2.41)
Eastern Sub-Saharan Africa	2381 (1673,3290)	5.42 (3.94,7.37)	6420 (4698,8440)	5.88 (4.31,7.76)	0.19 (0.16,0.22)
Central Sub-Saharan Africa	1358 (939,1882)	10.91 (7.66,14.91)	3475 (2345,4839)	11.11 (7.62,15.56)	-0.13 (-0.21,-0.05)
Western Sub-Saharan Africa	5652 (4084,7533)	9.19 (6.6,12.18)	14681 (10678,19067)	11.07 (8.07,14.43)	0.65 (0.54,0.75)

**Table 2:** DALYs and ASDR of ischemic stroke attributable to HFPG in 1990 and 2021, and trends over this period.

DALY	1990		2021		1990–2021
Location	DALYs cases No. (95% UI)	ASDR per 100,000 No. (95% UI)	DALYs cases No. (95% UI)	ASDR per 100,000 No. (95% UI)	EAPC in ASDR No. (95% CI)
Global	6189756 (4834685,7817249)	176.87 (138.36,222.99)	12371434 (9587506,15382662)	147.07 (113.94,183.06)	-0.72 (-0.81,-0.62)
Sex					
Male	2999824 (2340710,3767379)	198.74 (155.55,249.42)	6501754 (5078203,8096546)	172.65 (134.64,214.98)	-0.52 (-0.61,-0.44)
Female	3189932 (2472654,4014748)	159.6 (123.76,200.93)	5869681 (4471617,7374109)	125.64 (95.75,157.78)	-0.95 (-1.05,-0.84)
SDI					
Low SDI	232886 (171036,315514)	132.08 (97.64,177.75)	592442 (436513,779610)	144.78 (107.46,189.32)	0.24 (0.19,0.29)
Low-middle SDI	740615 (570946,952477)	149.26 (115.01,191.49)	2226720 (1661985,2814897)	175.42 (131.95,221.06)	0.51 (0.46,0.56)
Middle SDI	1473917 (1140334,1904192)	174.23 (134.76,224.68)	4073160 (3138040,5120243)	164.47 (126.91,206.4)	-0.16 (-0.25,-0.08)
High-middle SDI	2151025 (1686868,2686606)	242.67 (189.3,303.23)	3628252 (2827169,4540170)	183.54 (143.16,229.78)	-1.1 (-1.3,-0.9)
High SDI	1580162 (1228406,1951874)	139.17 (108.03,172.16)	1836729 (1418883,2258219)	78.48 (60.84,96.36)	-2.14 (-2.27,-2.01)
GBD region					
Oceania	3628 (2652,4770)	171.19 (126.9,223.02)	9346 (6862,12349)	167.16 (123.58,219.92)	-0.18 (-0.25,-0.12)
East Asia	1440623 (1091805,1897940)	201.52 (152.66,263.45)	4025618 (3048243,5158390)	193.32 (145.09,248.6)	0.05 (-0.19,0.3)
Southeast Asia	345665 (265369,441812)	171.87 (133.22,219.75)	1121063 (807247,1437371)	201.65 (146.44,258.51)	0.56 (0.43,0.68)
Central Asia	66267 (49886,83855)	154.09 (116.02,195.25)	149930 (114183,187822)	211.09 (162.26,263.64)	0.74 (0.49,1)
Central Europe	517326 (408002,639894)	370.74 (290.47,459.45)	560455 (438307,689393)	233.32 (182.46,286.89)	-1.82 (-1.97,-1.67)
High-income Asia Pacific	360907 (281784,445799)	197.06 (153.46,244.2)	390977 (293172,489339)	66.88 (51.35,83.71)	-3.88 (-4.04,-3.72)
Western Europe	832628 (636403,1039896)	134.04 (102.27,167.36)	587916 (442771,743950)	51.13 (38.71,64.75)	-3.31 (-3.45,-3.18)
Eastern Europe	737715 (568627,947310)	280.1 (216.29,359.23)	838016 (644310,1057787)	230.7 (177.18,291.11)	-1.28 (-1.71,-0.84)
Australasia	24070 (18405,30247)	103.9 (79.05,131.26)	28941 (21782,36076)	47.54 (36,59.4)	-2.72 (-2.83,-2.62)
Southern Latin America	59962 (45923,75361)	136.95 (104.7,171.95)	70070 (53383,86836)	76.82 (58.57,95.24)	-1.65 (-1.75,-1.54)
High-income North America	329431 (253137,414852)	88.57 (67.85,111.36)	573276 (438353,704788)	81.7 (63.09,100.31)	-0.69 (-0.92,-0.45)
Caribbean	35521 (27266,44217)	146.64 (113.04,181.96)	67525 (50154,85625)	124.57 (92.48,157.91)	-0.46 (-0.55,-0.36)
South Asia	571013 (423395,771644)	123.88 (92.69,166.06)	1778873 (1336969,2352326)	135.93 (102.58,178.33)	0.12 (0.06,0.18)
Central Latin America	83671 (66269,103584)	116.93 (92.2,144.75)	161974 (124790,203387)	68.09 (52.46,85.52)	-2.02 (-2.18,-1.86)
Andean Latin America	11983 (9068,15331)	66.06 (49.95,84.33)	30888 (22537,40082)	54.82 (39.93,71.01)	-0.74 (-0.88,-0.61)
Tropical Latin America	167315 (129979,209836)	214.98 (166.87,270.26)	253194 (197681,312038)	102.06 (79.48,125.92)	-2.21 (-2.35,-2.08)
North Africa and Middle East	386236 (294041,497211)	278.82 (213.1,358.12)	1132376 (854130,1412108)	290.32 (219.01,360.23)	0.14 (0.12,0.17)
Southern Sub-Saharan Africa	25605 (19022,32860)	113.94 (84.89,146.59)	85377 (66260,107270)	180.9 (139.56,227.8)	1.81 (1.34,2.29)
Eastern Sub-Saharan Africa	48755 (35009,67249)	91.38 (66.04,123.75)	131779 (97608,173758)	103.71 (77.04,136.73)	0.32 (0.29,0.36)
Central Sub-Saharan Africa	30877 (21803,41774)	193.38 (137.48,260.76)	75591 (52130,105092)	195.18 (135.99,267.23)	-0.15 (-0.23,-0.08)
Western Sub-Saharan Africa	110559 (79455,147717)	155.58 (112.58,206.78)	298248 (217676,387177)	192.5 (141.11,248.2)	0.72 (0.61,0.84)

**Figure 1:**
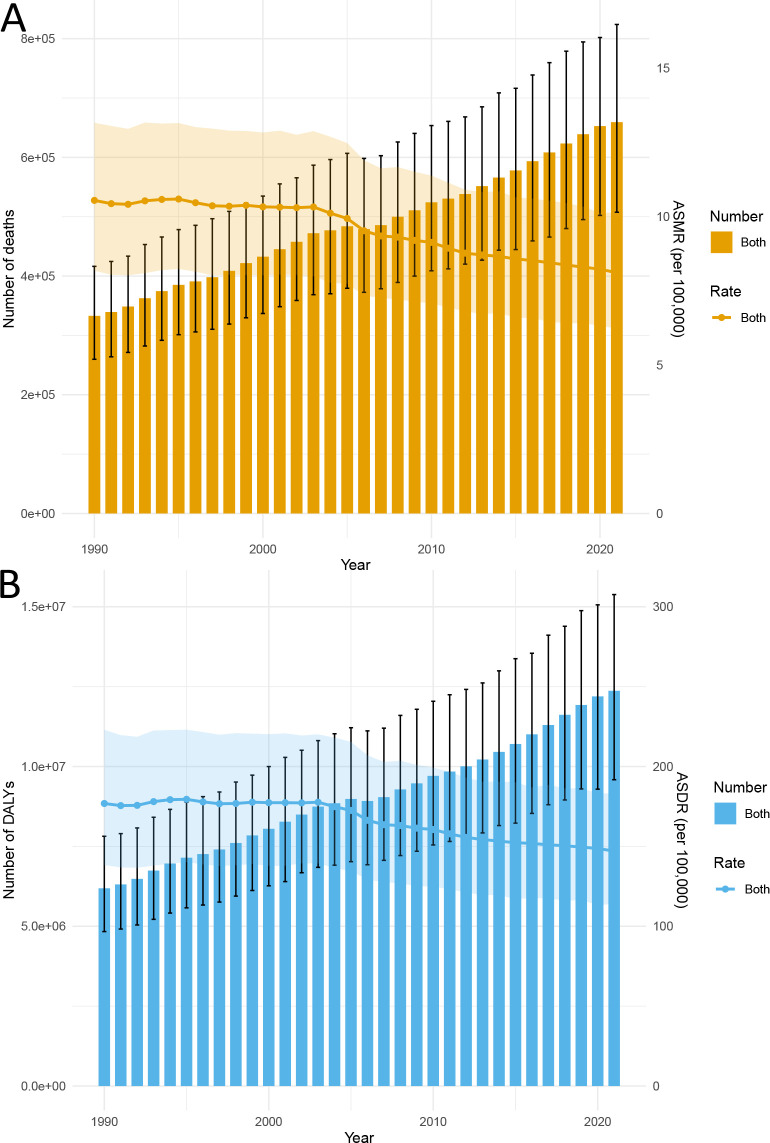
Trends in the global burden of ischemic stroke attributable to HFPG from 1990 to 2021: **(A)** Deaths and ASMR, **(B)** DALYs and ASDR.

The global ASMR and ASDR for ischemic stroke related to HFPG have shifted from 10.55 (95% UI: 8.18 to 13.16) per 100,000 in 1990 to 8.11 (95% UI: 6.23 to 10.14) per 100,000 in 2021, and from 176.87 (95% UI: 138.36 to 222.99) per 100,000 in 1990 to 147.07 (95% UI: 113.94 to 183.06) per 100,000 in 2021 (**Tables 1**, **2**). Consequently, while the absolute number of deaths and DALYs has doubled, the global ASMR and ASDR have exhibited a downward trend, with corresponding EAPCs of -0.96 (95% CI: -1.05 to -0.86) and -0.72 (95% CI: -0.81 to -0.62), respectively (**Figures 1A, B**, **Tables 1**, **2**).

### 3.2 Ischemic stroke burden attributable to HFPG by SDI regions

**Figure 2:**
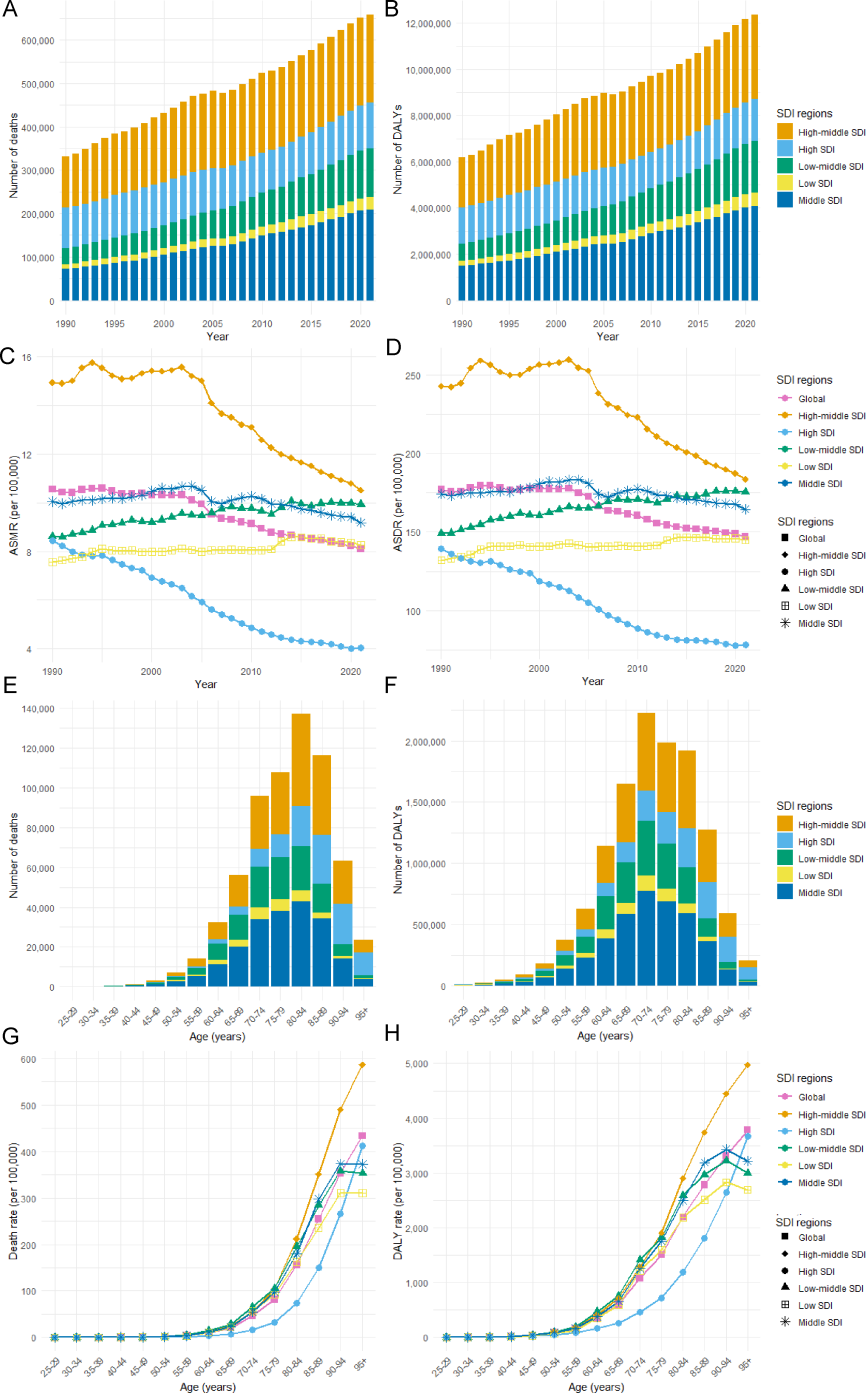
Ischemic Stroke Burden Attributable to HFPG by SDI Region. **(A)** Deaths, **(B)** DALYs, **(C)** ASMR, and **(D)** ASDR from 1990 to 2021. **(E)** Deaths, **(F)** DALYs, **(G)** Mortality Rate, and **(H)** DALY Rate by Age in 2021.

Between 1990 and 2021, the number of deaths and DALYs from ischemic stroke attributable to HFPG increased across all SDI regions. In 1990, the high-middle SDI region reported the highest deaths, with 118,568 (95% UI: 92,777 to 147,443), followed by the high SDI region and the middle SDI region (**Figure 2A**, **Table 1**). The low SDI region had the fewest deaths, totaling 11,045 (95% UI: 8,141 to 14,940). By 2021, the middle SDI region had the highest deaths at 208,524 (95% UI: 159,415 to 264,319), followed closely by the high-middle SDI region (**Figure 2A**, **Table 1**). The low SDI region remained the lowest with 28,790 (95% UI: 21,143 to 37,739). In 1990, the high-middle SDI region ranked first in DALYs, reporting 2,151,025 cases (95% UI: 1,686,868 to 2,686,606). By 2021, it had dropped to second place, with the middle SDI region taking the lead with 4,073,160 cases (95% UI: 3,138,040 to 5,120,243) (**Figure 2B**, **Table 2**). This shift indicates that while the majority of global deaths and DALYs were concentrated in regions with higher SDI levels, there has been a gradual transition towards areas with lower SDI levels.

The high-middle SDI region ranked first in both ASMR and ASDR in 1990 and 2021, respectively, while the lowest ASMR and ASDR regions transitioned from low SDI to high SDI. From 1990 to 2021, ASMR and ASDR showed significant variability in their developmental trends across various SDI regions. Regions with low and low-middle SDI showed rising ASMR and ASDR, whereas the rest saw a decrease. In the high SDI region, ASMR and ASDR experienced the most significant decrease, with EAPCs of -2.68 (95% CI: -2.82 to -2.54) and -2.54 (95% CI: -2.27 to -2.01), respectively. In contrast, the low-middle SDI region exhibited the highest increase, with EAPCs of 0.48 (95% CI: 0.42 to 0.54) for ASMR and 0.51 (95% CI: 0.46 to 0.56) for ASDR. Compared to the global average, ASDR was greater in the high-middle, low, and middle SDI regions. Conversely, all regions except the high SDI region had ASMR above the global average (**Figures 2C, D**).

In 2021, we observed significant differences in the distribution of HFPG-related ischemic stroke deaths and DALYs across various age groups. The majority of deaths in each SDI region were concentrated among individuals aged 80 to 84, whereas the highest DALYs were predominantly found in the age group of 70 to 74 (**Figures 2E, F**). Furthermore, mortality and DALY rates generally exhibited an upward trend with age. Specifically, the mortality rate escalated rapidly after the age of 55, while the DALY rates showed a marked increase after the age of 50 (**Figures 2G, H**).

### 3.3 Ischemic stroke burden attributable to HFPG by 21 GBD regions

In 2021, the deaths and DALYs in East Asia, South Asia, North Africa and Middle East were among the highest (**Tables 1**, **2**). Conversely, Oceania reported the lowest figures, with deaths at 398 (95% UI: 291 to 536) and DALYs at 9,346 (95% UI: 6,862 to 12,349). The regions with the highest ASMR were North Africa and the Middle East [95% UI: 16.83 (12.74 to 21.13)], Central Europe [95% UI: 14.3 (11.07 to 17.76)], and Eastern Europe [95% UI: 13.64 (10.36 to 17.28)], while Australasia had the lowest ASMR at 2.73 (95% UI: 1.98 to 3.45) (**Table 1**). The highest and lowest regions of ASDR align with those of the ASMR (**Table 2**).

From 1990 to 2021, the ASMR decreased in 14 regions, while 7 regions experienced an increase. Southern Sub-Saharan Africa experienced the greatest growth, with an EAPC of [95% CI: 1.9 (1.39 to 2.41)], followed by Central Asia, and Western Sub-Saharan Africa (**Table 1**). In contrast, the region with the largest decline was the High-Income Asia-Pacific, with an EAPC of [-4.56 (95% CI: -4.71 to -4.4)], followed by Western Europe and Australasia (**Table 1**). Regarding the ASDR, 13 regions demonstrated a downward trend, whereas 8 regions showed an increase. Among the 21 regions analyzed, the regions showing the greatest increases and decreases in ASDR were the same as those for ASMR (**Table 2**).

### 3.4 Ischemic stroke burden attributable to HFPG by 204 countries and territories

**Figure 3:**
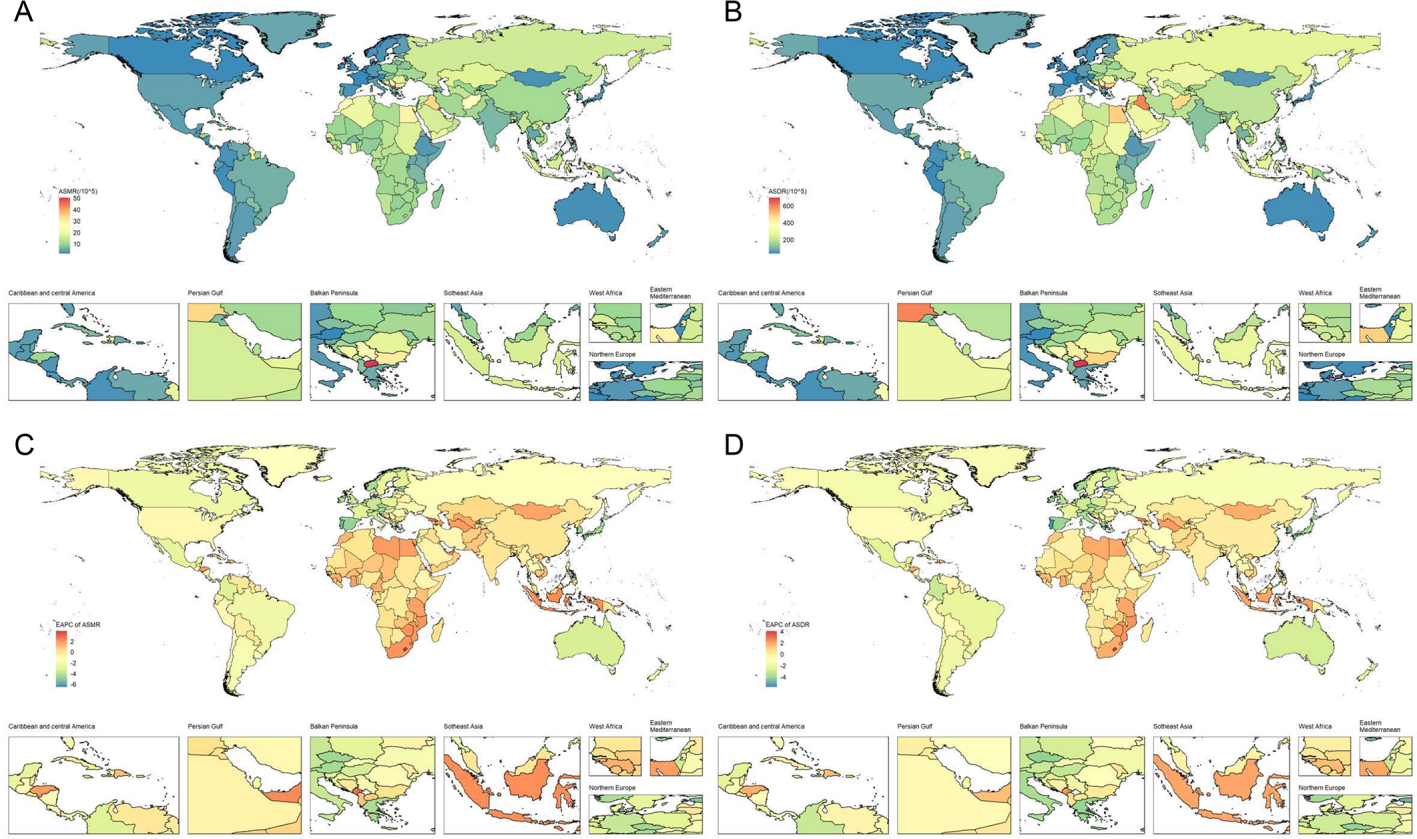
The spatial distribution of ischemic stroke attributable to HFPG in 2021. **(A)** ASMR, **(B)** ASDR, **(C)** the EAPC of ASMR, and **(D)** the EAPC of ASDR.

In 2021, China, India, and Russia had the highest HFPG-related ischemic stroke mortality (**Supplementary Table S1**). The order of DALYs was consistent with the mortality ranking, with China, India, and Russia maintaining their positions (**Supplementary Table S2**). North Macedonia, Iraq, and Bulgaria exhibited the highest ASMR, recorded at 50.36 (95% UI: 36.42 to 63.75), 34.8 (95% UI: 25.35 to 45.14), and 30.66 (95% UI: 23.32 to 38.11), respectively (**Figure 3A**, **Supplementary Table S1**). In comparison, the highest ASDR were recorded for North Macedonia, Iraq, and Egypt, with rates of 696.45 (95% UI: 508.93, 882.3), 597.85 (95% UI: 429.66, 782.31), and 495.62 (95% UI: 362.45, 660.66), respectively (**Figure 3B**, **Supplementary Table S1**). Conversely, Singapore had the lowest ASMR at 1.59 (1.18, 1.99), while France had the lowest ASDR at 34.56 (95% UI: 25.29, 44.36) (**Supplementary Table S2**).

A total of 80 countries exhibited a growing trend in ASMR, with the most significant increase observed in Lesotho at 3.9 (95% CI: 3.33 to 4.47), followed by Georgia and Montenegro (**Supplementary Table S1**). In contrast, Singapore experienced the most significant decrease, showing an EAPC of -6.49 (95% CI: -6.98 to -6) (**Figure 3C**, **Supplementary Table S1**). Similarly, 84 countries experienced an increase in ASDR. The countries with the highest growth in ASDR were the same as those for ASMR, including Lesotho at 4.07 (95% CI: 3.53 to 4.61), Georgia at 2.67 (95% CI: 2.18 to 3.16), and Montenegro at 2.27 (95% CI: 2.09 to 2.46). Singapore again reported the most significant decline, showing an EAPC of -5.72 (95% CI: -5.94 to -5.49) (**Figure 3D**, **Supplementary Table S2**).

### 3.5 Global burden of Ischemic stroke attributable to HFPG by age and gender

**Figure 4:**
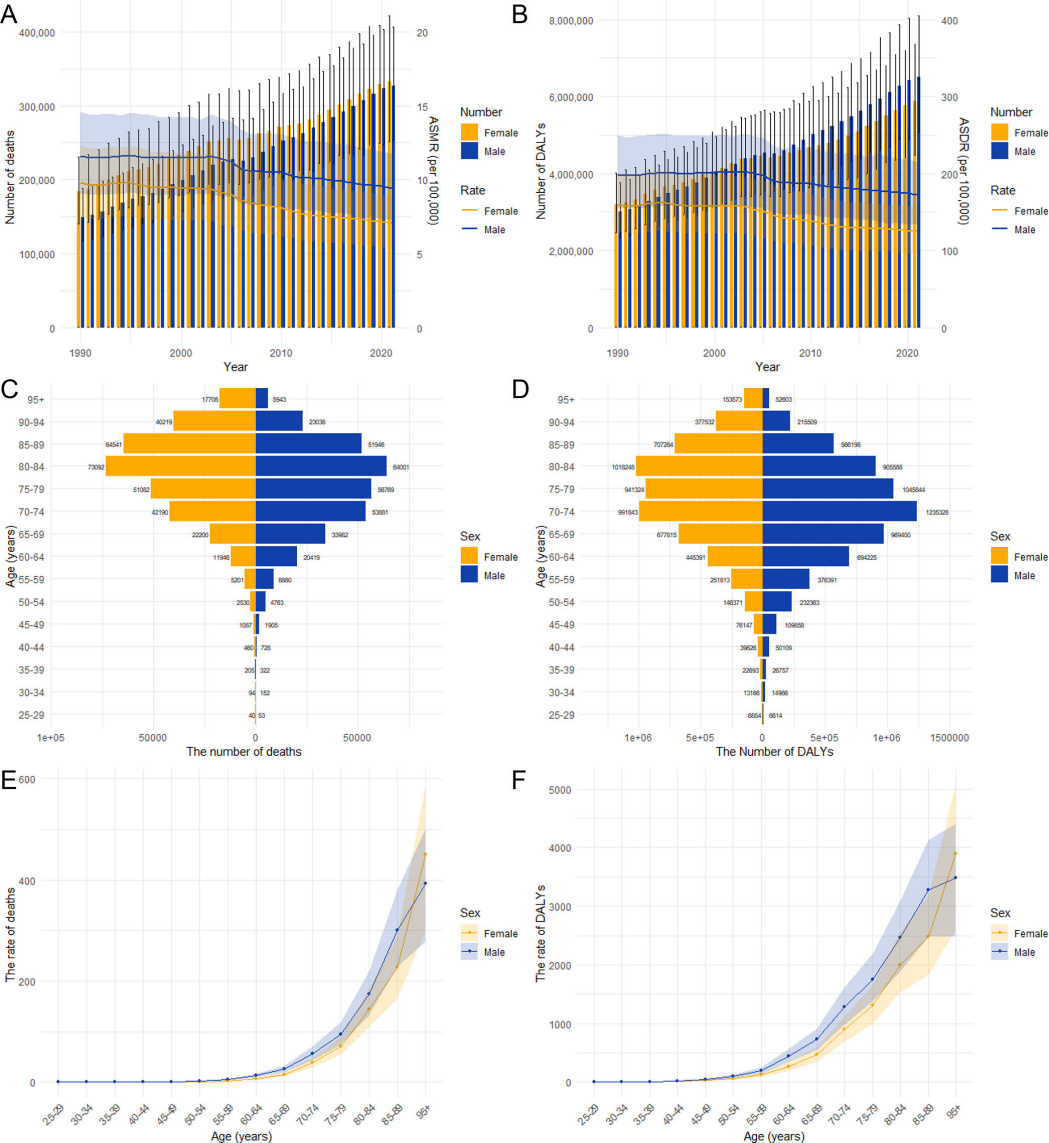
The burden of ischemic stroke attributable to HFPG by age and gender. The number of deaths and ASMR **(A)** due to ischemic stroke attributed to HFPG, as well as DALYs and ASDR **(B)**, by gender from 1990 to 2021. The age-specific number of deaths **(C)**, DALYs **(D)**, mortality rates **(E)**, and DALY rates **(F)** of Ischemic stroke attributable to HFPG by gender in 2021.

From 1990 to 2021, the ASMR and ASDR caused by HFPG-attributable ischemic stroke generally decreased each year for both sexes. Throughout this period, the ASMR and ASDR have consistently been higher in men compared to women. Despite the annual decline in ASMR and ASDR, the overall number of deaths and DALYs resulting from HFPG-attributable ischemic stroke in both men and women has continued to increase annually. Notably, the deaths among women have consistently exceeded that of men, although this gap has been narrowing over time. In contrast, the DALYs in men have surpassed that of women, with the disparity appearing to widen each year (**Figures 4A, B**).

In 2021, the global burden of HFPG-attributable ischemic stroke primarily affected middle-aged and elderly individuals. Mortality and DALY rates rose with age, especially for individuals over 50, affecting both men and women. Except for individuals aged over 93, mortality and DALY rates have consistently been higher in men compared to women (**Figures 4E, F**). The highest number of deaths from HFPG-attributable ischemic stroke occurred within the 80–84 age group for both genders. Furthermore, the peak number of DALYs was observed at ages 80–84 for women and 70–74 for men, respectively (**Figures 4C, D**).

### 3.6 Relationship between SDI and HFPG–related Ischemic stroke burden

**Figure 5:**
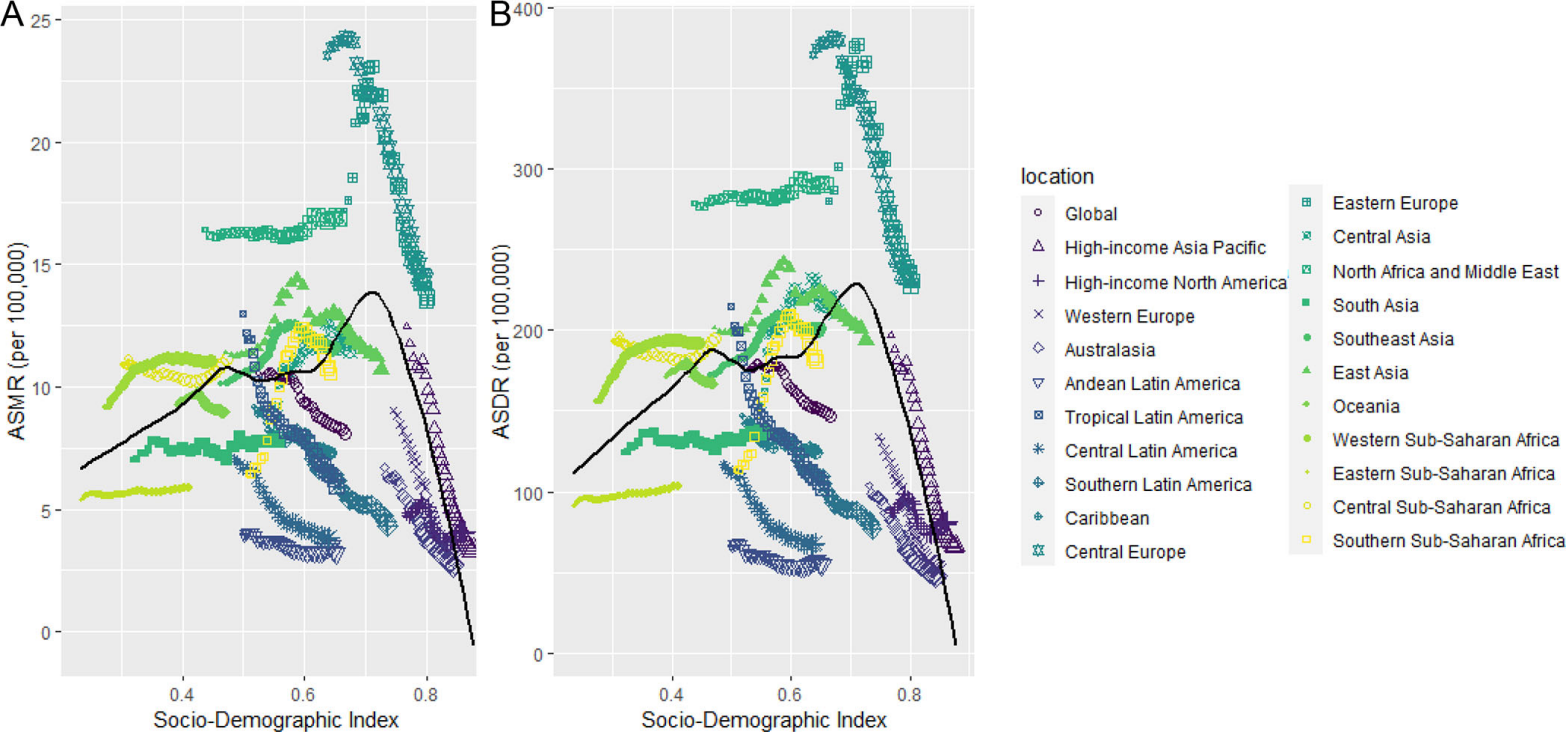
Trend in age-standardized rates of ischemic stroke attributable to HFPG for 21 GBD regions by SDI, 1990–2021. **(A)** Deaths. **(B)** DALYs.

**Figure 6:**
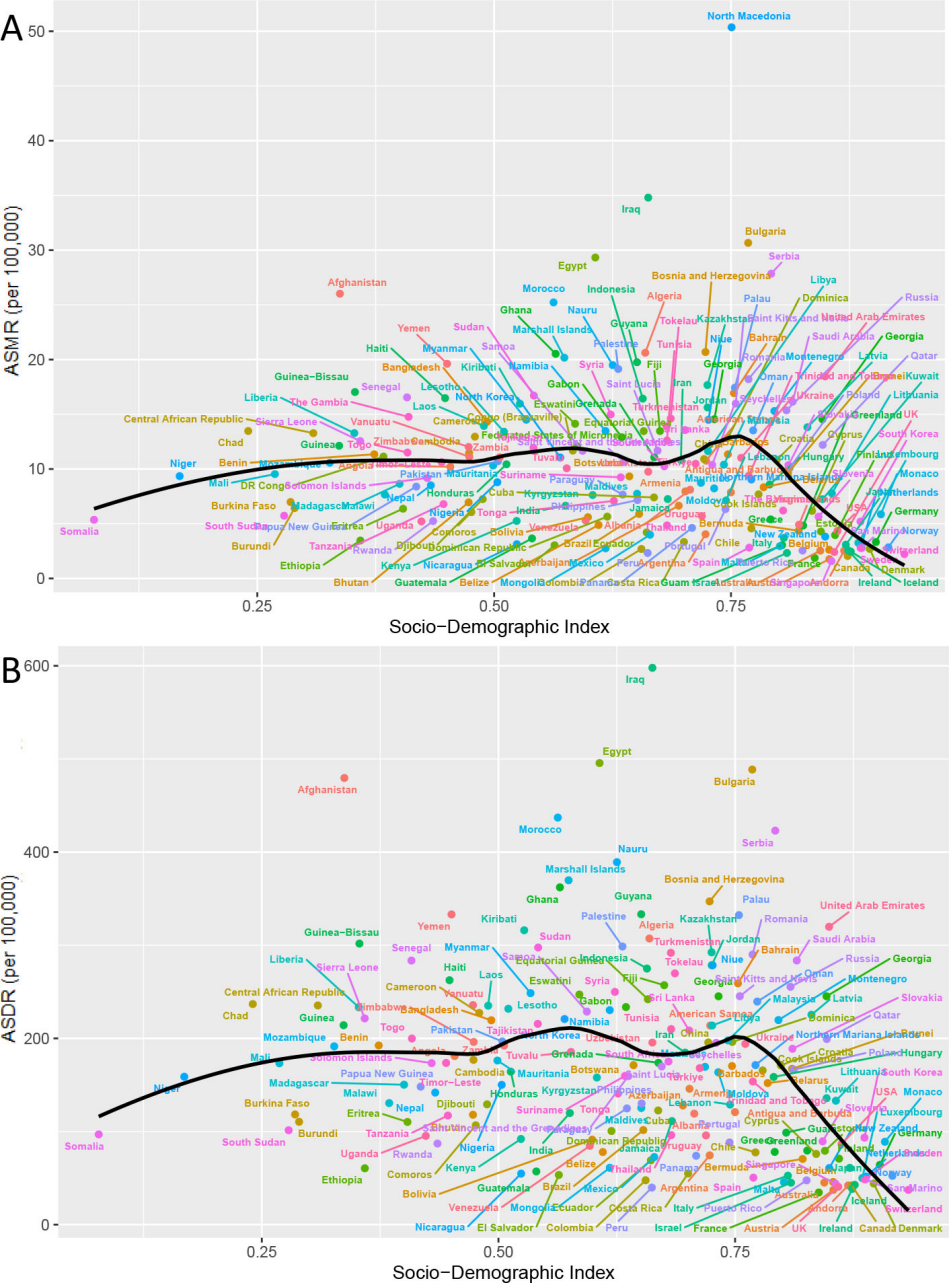
Trend in age-standardized rates of ischemic stroke attributable to HFPG for 204 countries and territories by SDI, 1990–2021. **(A)** Deaths. **(B)** DALYs.

The relationship between the SDI and ASDRs of HFPG-related ischemic stroke closely paralleled that of SDI and ASMRs, exhibiting distinct nonlinear trends. When SDI was less than 0.45, both ASDRs and ASMRs demonstrated a gradual increasing trend. In the range of SDI between 0.45 and 0.62, ASDRs and ASMRs exhibited steady fluctuations. Beyond an SDI of 0.62, there was a period of rapid growth, peaking at an SDI of 0.71, after which both values sharply declined (**Figures 5A, B**). At the national level, ASMRs and ASDRs reflected a similar trend. When the SDI was below 0.75, both values were characterized by volatile nonlinear growth. However, once the SDI surpassed 0.75, both values declined rapidly (**Figures 6A, B**).

In addition, we further analyzed health inequalities related to the SDI. The absolute values of SII for DALYs and deaths were 122.34 and 7.59, respectively, in 1990, and 120.95 and 7.66, respectively, in 2021 (**Supplementary Figures S2A, C**). The concentration index of relative inequality in DALYs and deaths were 0.17 and 0.22, respectively, in 1990, and 0.10 and 0.12, respectively, in 2021 (**Supplementary Figures S2B, D**). These findings indicate that while health inequalities have somewhat improved, the disease burden remains concentrated in wealthier regions.

### 3.7 Decomposition analysis

**Figure 7:**
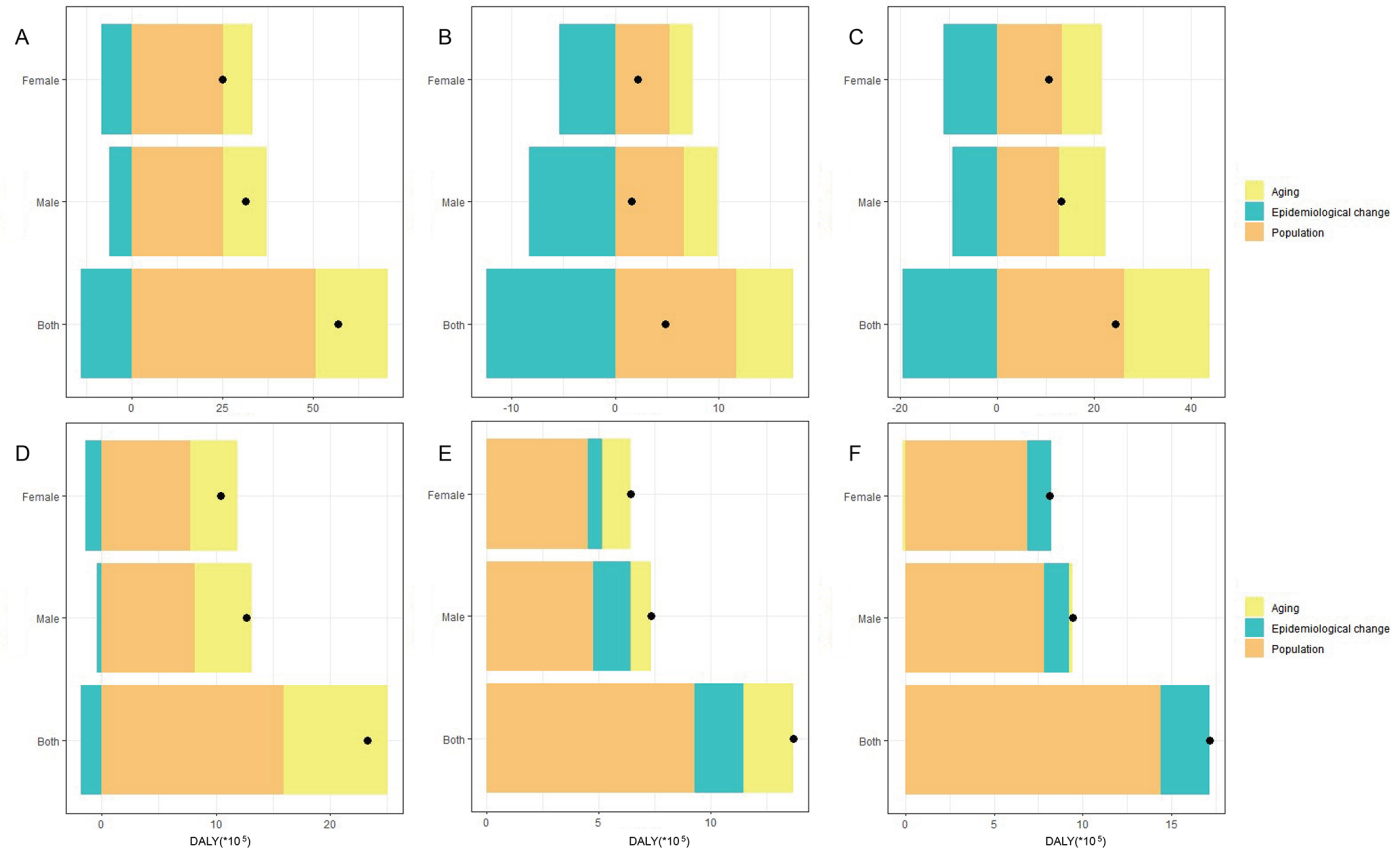
Decomposition analysis of DALY indicators for ischemic stroke attributed to HFPG in different SDI regions from 1990 to 2021. **(A)** Global. **(B)** High SDI. **(C)** High-middle SDI. **(D)** Middle SDI. **(E)** Low-middle SDI. **(F)** Low SDI.

To investigate the relative contributions of population growth, aging, and epidemiological changes to disease, we performed a decomposition analysis of DALYs and deaths from ischemic stroke attributable to HFPG on a global scale and across five SDI quantiles. The results indicate that, from 1990 to 2021, population growth emerged as the primary driving factor for the increase in global DALYs, contributing 89.29%. Aging was the second most significant factor, accounting for 35.19%. Notably, the largest contributions to DALY growth were observed in the high SDI quintiles, with population growth and aging contributing 243% and 114.22%, respectively. Conversely, the impact of epidemiological changes on DALYs was negative on a global scale, with a contribution rate of -24.48%. Although most regions exhibited negative growth due to epidemiological changes, the low-middle and low SDI quintiles demonstrated notable positive growth, with 15.88% and 16.02% contribution rates, respectively (**Figure 7**, **Supplementary Table S3**).

Globally, population growth contributed more to the increase in DALYs among women than among men, while aging had a greater impact on the growth of DALYs in men than women. Notably, in both the high SDI and low SDI regions, the contribution rate of aging to the growth of DALYs was higher for men than women. When excluding the High SDI regions, population growth in the remaining SDI regions showed a greater contribution rate for women than for men. The impact of epidemiological changes on negative global gains in DALYs was more significant among women than men (**Figure 7**, **Supplementary Table S3**). Furthermore, the decomposition analysis of deaths paralleled that of DALYs. Population growth and aging were the primary factors contributing to this increase, accounting for 93.7% and 42.67%, respectively. In contrast, epidemiological changes exhibited a negative growth rate, contributing -36.37%. (**Supplementary Figure S1**, **Supplementary Table S3**).

### 3.8 Global disease burden prediction for Ischemic stroke attributable to HFPG

**Figure 8:**
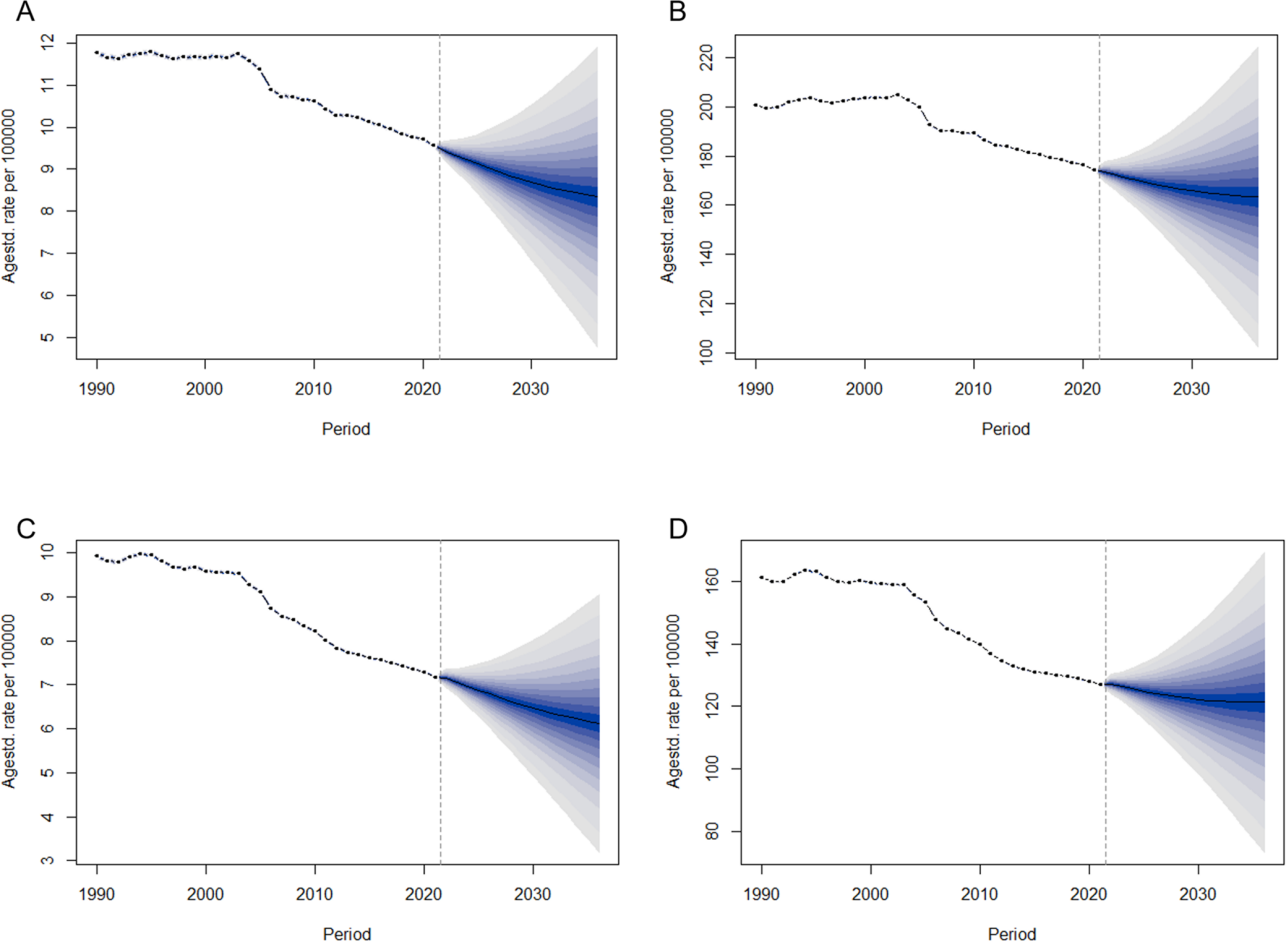
Trends in ASMR and ASDR for ischemic stroke due to HFPG by sex, globally from 1990 to 2036, based on the BAPC model. **(A)** ASMR of male. **(B)** ASDR of male. **(C)** ASMR of female. **(D)** ASDR of female.

It is predicted that over the next 15 years, the mortality and DALY rates associated with ischemic stroke caused by HFPG will decline for both men and women. However, compared to the decrease in mortality, the DALY rates for both genders are expected to plateau by 2036 (**Figure 8**). Furthermore, by 2036, the ASMR and the ASDR will remain higher in men than women. Specifically, the ASMR for men is forecasted to decrease from 9.57 per 100,000 in 2021 to 8.34 per 100,000 in 2036, while their ASDR is expected to decline from 174.29 per 100,000 in 2021 to 163.30 per 100,000 in 2036 (**Figures 8A, B**). In contrast, the ASMR for women is anticipated to fall from 7.19 per 100,000 in 2021 to 6.13 per 100,000 in 2036, and their ASDR is projected to decrease from 127 per 100,000 in 2021 to 121.33 per 100,000 in 2036 (**Figures 8C, D**).

## 4 Discussion

This study provided a comprehensive and systematic analysis of the disease burden of ischemic stroke attributable to HFPG. Our findings indicated that the global deaths and DALYs from ischemic stroke related to HFPG were nearly double in 2021 compared to 1990. However, the ASMR and ASDR exhibited a downward trend. Furthermore, the disease burden of ischemic stroke due to HFPG has been progressively shifting from higher to lower SDI regions. Regarding gender classification, men experienced a significantly higher burden compared to women, though the disease burden has been decreasing for both sexes. Notably, the decline in ASMR and ASDR values was more pronounced in women. It is important to highlight that the disease burden remains relatively severe among middle-aged and elderly populations. Analysis of decomposition indicated that the primary factors contributing to the rise in the global disease burden were population growth and aging.

A strong link exists between hyperglycemia and ischemic stroke. Hyperglycemia activates various molecular mechanisms through oxidative stress and other pathways, which disrupt the structure and function of endothelial cells, ultimately compromising the integrity of the blood-brain barrier. This disruption is a critical pathological mechanism underlying cerebral vascular damage (23). These molecular processes are frequently accompanied by inflammatory responses, resulting in elevated levels of inflammatory markers such as IL-6, further exacerbating endothelial injury and promoting atherosclerosis (24). Furthermore, hyperglycemia heightens the risk of thrombosis by inducing platelet dysfunction and disrupting the coagulation cascade (25). Following the onset of ischemic stroke, hyperglycemia can lead to brain edema through the activation of the NLRP3 inflammasome, worsening neurological outcomes and increasing rates of mortality and disability (26). Notably, HFPG, along with other metabolic factors such as low-density lipoprotein and high body mass index, frequently interacts to influence the progression of stroke. For example, obesity and hyperglycemia can interact through factors such as adiponectin, free fatty acids, and gut microbiota, synergistically enhancing insulin resistance and thereby increasing the risk of stroke (27, 28). Therefore, managing blood glucose levels is essential to lessen the disease burden for those at high risk of ischemic stroke.

The disease burden of ischemic stroke related to HFPG varies significantly across different SDI levels. Notably, high SDI regions, including High-income Asia Pacific, Western Europe, and Australasia, have well-documented achievements in reducing the disease burden. This success can be attributed to a robust healthcare system, advanced medical technology, adequate pharmaceutical resources, a strong emphasis on health education, and effective allocation of funds. Representative countries include France, Singapore, and Australia. Singapore has successfully increased public awareness of diabetes to 82.7% through initiatives such as the “War on Diabetes” campaign (29). The revised Food-Based Dietary Guidelines (FBDG) introduced in France in 2017 have enhanced dietary habits and decreased the risk of type 2 diabetes by 49% (30). Additionally, Australia has established a community-based primary health care system through Medicare, achieving universal health coverage and enhancing the management of chronic diseases (31). Despite these significant advancements, there remains room for improvement in these nations to further alleviate the disease burden. Potential measures include enhancing the personalization of disease management, developing more targeted new drugs or testing methodologies, promoting multidisciplinary collaboration to upgrade comprehensive disease management services, and strengthening the application of digital health technologies (such as remote monitoring and data analysis) to enhance disease prediction and management efficiency (32–35).

The high-middle and middle SDI regions currently represent the primary sources of disease burden. However, the disease burden in the low-middle and low SDI regions is steadily increasing. Particular attention should be directed towards two populous countries, China in East Asia and India in South Asia, which report the highest deaths and DALYs. This trend is primarily driven by population growth, accelerated industrialization, and a westernized lifestyle associated with economic development (36). Therefore, in these two countries, the relationship between hyperglycemia and the risk of ischemic stroke should be publicized through various channels, such as social media and community activities, to enhance public awareness of blood sugar levels. Furthermore, it is essential to strengthen blood sugar screening and management across multiple dimensions, including implementing community-based screening programs, which can yield significant social and economic benefits. Regarding countries, North Macedonia exhibited the highest burden of ASMR and ASDR. The nation is currently undergoing a phase of social transition, during which poor dietary habits—characterized by the consumption of flour bread, inexpensive animal products (pork, beef, and fatty cheese), and excessive use of sunflower oil—have significantly contributed to this severe health burden. Therefore, it appears particularly beneficial for North Macedonia to advocate for adopting a Mediterranean diet in this context (37, 38). Furthermore, it is crucial to prioritize blood glucose screening among women and rural municipalities populations within the country (39). In 2021, North Africa and Middle East exhibited the highest ASMR and ASDR. Southern Sub-Saharan Africa, Central Asia, and Western Sub-Saharan Africa experienced the most significant increases in ASMR and ASDR. Research indicates a significant shortage of diabetes treatment and diagnosis in Africa. The availability of premixed insulin stands at 49.4%, while metformin is available at 47.0%. Blood glucose meters are relatively well supplied at 49.5%, in contrast to the HbA1c test, which is only available at 24.6% (40). Sub-Saharan Africa faces a deficiency in diabetes education. Although many individuals are aware of the primary risk factors for diabetes, they tend to underestimate their own weight, resulting in a general lack of engagement in weight management and healthy dietary practices (41). Moreover, the African region is grappling with the dual challenges of nutritional transition and malnutrition, further complicating efforts to manage blood glucose levels and reduce the risk of stroke (42). Therefore, in addition to actively seeking medical assistance from developed regions, these regions must enhance public health awareness, improve diet and lifestyle, strengthen screening for high-risk groups, and develop appropriate medical policies. Furthermore, non-drug therapies such as acupuncture, yoga, and Tai Chi can serve as complementary approaches in resource-poor areas (43–46); however, further evidence-based research is necessary to support their efficacy (47). In addition to economic factors, race seems to influence the disease burden. A recent study demonstrated that, following intensive glycemic control in patients with ischemic stroke and hyperglycemia, Black patients are more likely to experience poor prognoses compared to their White counterparts (48). Consequently, countries must consider various national contexts, including racial disparities, when developing disease prevention and management strategies. Recent studies increasingly indicate that fluctuations in blood sugar levels have a more significant impact on ischemic stroke than simple hyperglycemia. For instance, research has demonstrated that blood sugar fluctuations can exacerbate cognitive impairment following a stroke (49). On the other hand, the impact of stress hyperglycemia on ischemic stroke has received increasing attention (50). Therefore, how to further refine blood sugar management has become an important issue.

Interestingly, while health inequality analysis indicates a decline in the overall level, the burden remains concentrated in affluent areas. This phenomenon is primarily the result of the dual imbalance in risk exposure and medical resource allocation that accompanies globalization. In high SDI regions, long-term adherence to Western dietary patterns, the widespread availability of processed foods, and sedentary work environments have contributed to an increased risk of HFPG (51). Concurrently, as the population ages and grows, the reliance on advanced medical technology to convert acute deaths into chronic disabilities has led to an increase in the reported statistics of DALYs. In low SDI regions, the issues of hidden hunger and metabolic disorders that arise during the nutritional transition have not been adequately addressed by primary health systems (42). Additionally, the lack of capacity for post-stroke treatment, which contributes to premature mortality, may further obscure the true burden due to missed diagnoses and reporting mechanisms.

Significant age differences exist among ischemic stroke patients, particularly related to HFPG. Currently, middle-aged and elderly individuals, especially those over 50 years old, represent the most vulnerable populations, with their mortality and DALY rates increasing with age. In comparison to younger individuals, middle-aged and elderly patients are more likely to experience a more significant disease burden due to factors such as vascular aging, decline in endothelial function, metabolic disorders, and long-term unhealthy lifestyle choices, including prolonged periods of inactivity and smoking (52–54). Consequently, patients with high-risk factors for ischemic stroke should prioritize fasting blood glucose screening after the age of 50. Furthermore, given the increasing incidence of diabetes among younger individuals, it is recommended that countries adjust the age threshold for screening following prevailing conditions (55). Early screening also aids in identifying individuals with prediabetes, facilitating timely intervention and management of the condition.

The current disease burden of ischemic stroke attributed to HFPG is significantly more significant in men than in women. Men are generally more susceptible to insulin resistance due to gender differences in sex hormones, fat distribution, and inflammatory responses (56). Abnormal androgen levels may decrease insulin sensitivity and impair glucose utilization efficiency in men (57). Furthermore, diabetic men often exhibit lower testosterone levels, which increases the risk of hypercoagulation and the likelihood of stroke (58). Typically, men possess a higher proportion of visceral fat and secrete more pro-inflammatory factors such as IL-6 and TNF-α, which promote insulin resistance and atherosclerosis (56). In contrast, women have more subcutaneous fat. However, after menopause, the increase in visceral fat heightens the risk of HFPG (56). Additionally, a study reports that in 2020, the male smoking rate was five times that of females, with smoking potentially increasing the risk of insulin resistance by 2.19 times (59, 60). Men generally consume more alcohol than women. Excessive drinking impairs β-cell function, worsens insulin resistance, and increases the risk of diabetes and cardiovascular events (61). On the other hand, insufficient awareness of healthcare leads to delayed medical visits and poor compliance among men (62). A South Korean study revealed gender differences in health awareness, with males having an 8.2% lower diabetes awareness rate (60.2%) compared to females (68.4%). Nevertheless, the disease burden among women should not be overlooked in the short term, as more women than men currently die from this condition. Of particular concern are postmenopausal women, as the burden of ischemic stroke related to HFPG is expected to rise due to declining estrogen levels (63). Research indicates that estrogen may reduce insulin resistance through the miR-10a/b-5p/NCOR2 pathway, inhibit the release of pro-inflammatory cytokines to regulate immune responses, and play a neuroprotective role (64, 65). However, after menopause, its metabolic and cardiovascular protective effects are diminished.

Surprisingly, significant progress has been made in addressing the disease burden of ischemic stroke associated with HFPG. As the predictive analysis shows, the disease burden will show a clear downward trend over the next 15 years. Notably, the decline in ASMR is more pronounced than that of ASDR. This suggests that while advancements in the treatment of ischemic stroke related to HFPG are crucial, the importance of chronic disease management should also be increasingly emphasized.

However, our study has certain limitations. First, the GBD database relies on the differentiated reporting systems of various countries. In low SDI regions, underreporting occurs due to imperfect health monitoring systems; for instance, it has been reported that the proportion of undiagnosed diabetes in Africa reaches 54% (66). Even in high SDI regions such as the UK, there remains approximately a 30% risk of underreporting due to insufficient screening or the presence of asymptomatic hyperglycemia (67). Despite existing data limitations, the GBD database integrates multiple data sources and standardizes uncertainty intervals for data correction, thereby providing a reliable basis for cross-regional comparisons. Secondly, the BAPC model’s predictive assumption is that future medical intervention strategies will remain unchanged. However, the introduction of new therapies or medical policies, such as the widespread application of GLP-1 agonists (68), may alter the trajectory of diabetes epidemiology, resulting in a lower actual burden than currently predicted. Finally, although this study attempts to exclude the influence of other risk factors, single attribution typically cannot fully account for the occurrence and progression of diseases. For example, there may be a synergistic effect between high fasting blood glucose and obesity, making it challenging for this study’s algorithm to completely eliminate this impact. Therefore, future research should focus on developing hybrid models that integrate real-time intervention data with multi-risk interaction analysis to enhance prediction accuracy and the policy guidance value.

## 5 Conclusions

From 1990 to 2021, the global disease burden of ischemic stroke attributable to HFPG has decreased, particularly in the high SDI region. However, this burden remains substantial in lower SDI countries, and there is a notable shift towards regions with lower SDI. Furthermore, it is important to emphasize men, along with middle-aged and older adults, who are especially impacted by ischemic stroke associated with HFPG. Although a decline in this global burden is anticipated over the next 15 years, proactive strategies can still be implemented to mitigate the disease burden further. These strategies may include improving lifestyle, enhancing multidisciplinary disease management, and utilizing telemedicine. The findings of this study carry significant implications for policymakers, aiding in the evaluation and development of healthcare strategies.

## Data Availability

The datasets presented in this study can be found in online repositories. The names of the repository/repositories and accession number(s) can be found in the article/**Supplementary Material**.
