# Supplementary material for: The persistent challenge of ischemic stroke burden from high fasting plasma glucose: a global perspective
Source: Front Endocrinol (Lausanne). 2025 May 6;16:1490428. doi: 10.3389/fendo.2025.1490428 (PMC12088946; doi:10.3389/fendo.2025.1490428)
Supplement: Supplementary file 1 [file DataSheet1.pdf]

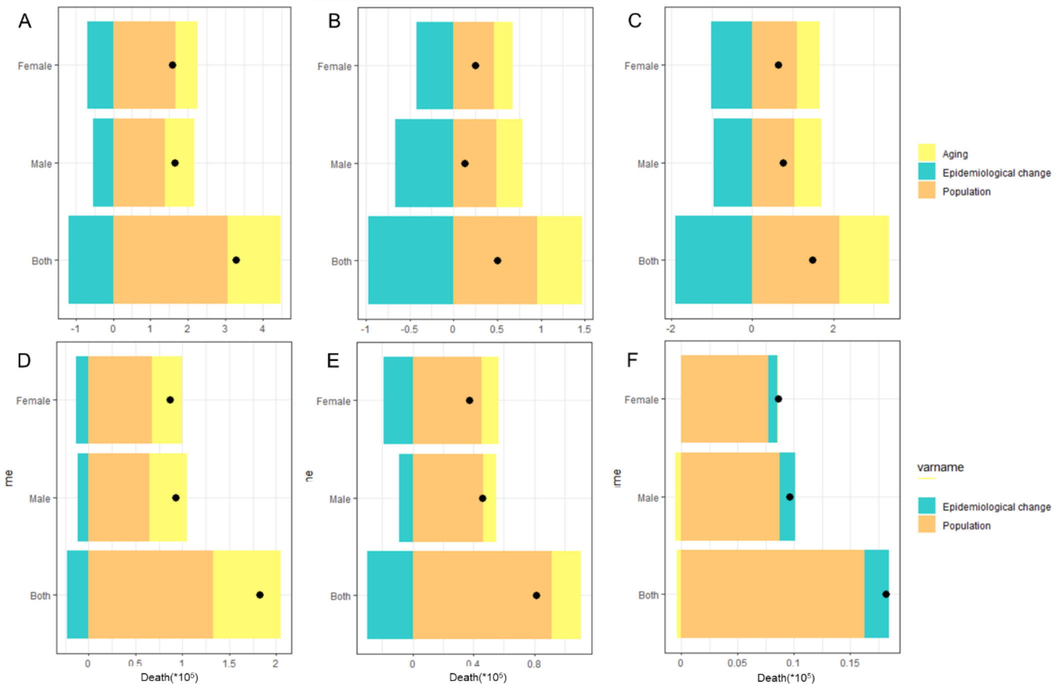

Supplementary Figure S1 Decomposition analysis of mortality indicators attributed to HFPG in ischemic stroke in different SDI regions from 1990 to 2021 (A) Global. (B) High SDI. (C) High-middle SDI. (D) Middle SDI. (E) Low-middle SDI. (F) Low SDI.
