# Supplementary material for: The persistent challenge of ischemic stroke burden from high fasting plasma glucose: a global perspective
Source: Front Endocrinol (Lausanne). 2025 May 6;16:1490428. doi: 10.3389/fendo.2025.1490428 (PMC12088946; doi:10.3389/fendo.2025.1490428)
Supplement: Supplementary file 2 [file DataSheet2.pdf]

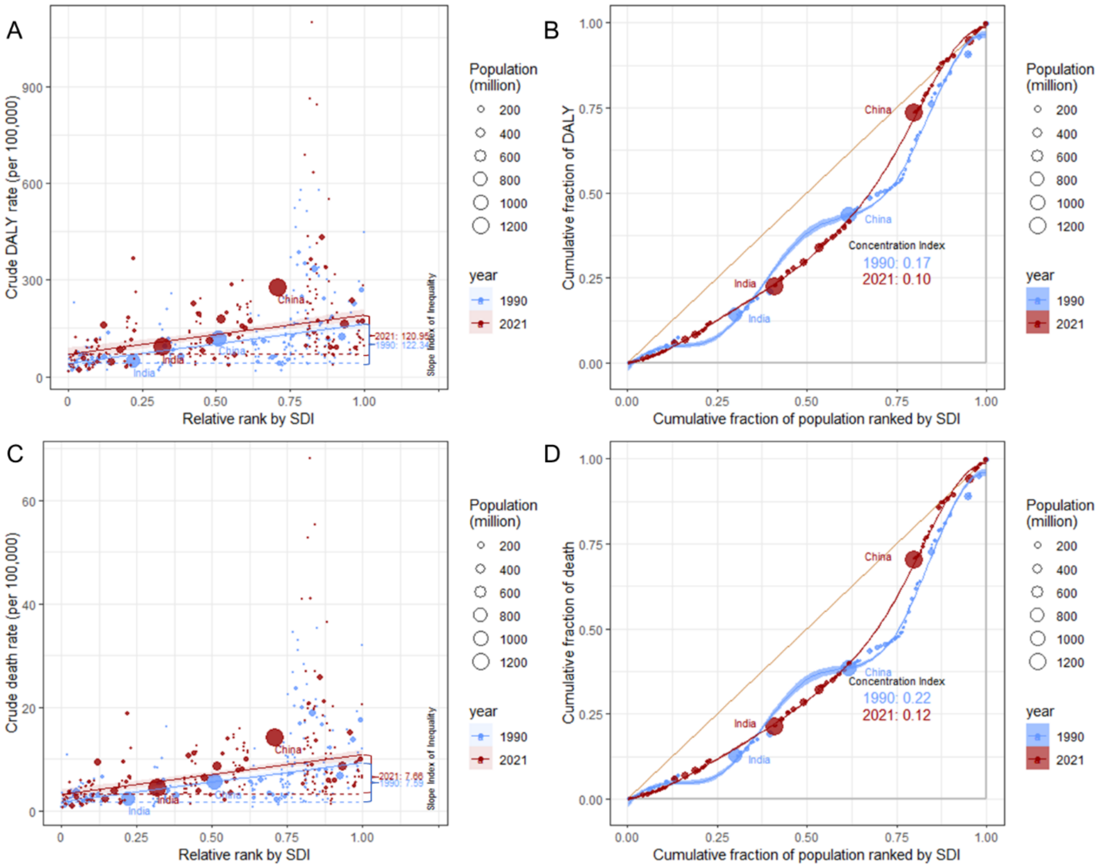

Supplementary Figure S2 SDI-related health inequality regression (A) and concentration (B) curves for global DALYs and SDI-associated health inequality regression (C) and concentration (D) curves for global deaths from 1990 to 2021.
