# Supplementary material for: The persistent challenge of ischemic stroke burden from high fasting plasma glucose: a global perspective
Source: Front Endocrinol (Lausanne). 2025 May 6;16:1490428. doi: 10.3389/fendo.2025.1490428 (PMC12088946; doi:10.3389/fendo.2025.1490428)
Supplement: Supplementary file 3 [file Table1.docx]

Table S1 Deaths and ASMR of ischemic stroke attributable to HFPG in 204 countries and territories in 1990 and 2021, and the temporal trends from 1990 to 2021.

| **Deaths** | 1990 | | 2021 | | 1990–2021 |
| --- | --- | --- | --- | --- | --- |
| Location | Deaths cases  No. (95% UI) | ASMR per 100,000  No. (95% UI) | Deaths cases  No. (95% UI) | ASMR per 100,000  No. (95% UI) | EAPC in ASMR  No. (95% CI) |
| Afghanistan | 1073 (736,1537) | 20.15 (14.14,28.69) | 1753 (1172,2455) | 26.02 (17.97,35.52) | 0.77(0.68,0.87) |
| Albania | 106 (76,141) | 6.89 (4.93,9.14) | 329 (222,461) | 8.11 (5.5,11.27) | 1.01(0.76,1.25) |
| Algeria | 1440 (1041,1926) | 21.8 (15.79,28.76) | 4837 (3372,6470) | 20.62 (14.91,27.43) | 0.07(0,0.14) |
| American Samoa | 2 (1,2) | 11.57 (8.89,14.6) | 4 (3,5) | 10.76 (7.92,13.83) | -0.51(-0.67,-0.35) |
| Andorra | 2 (1,2) | 3.94 (2.62,5.5) | 5 (3,6) | 2.54 (1.65,3.53) | -1.17(-1.42,-0.92) |
| Angola | 187 (133,263) | 8.85 (6.39,12.26) | 669 (469,906) | 10.21 (7.23,14.08) | 0.21(0.11,0.31) |
| Antigua and Barbuda | 7 (5,8) | 10.8 (8.2,13.63) | 7 (5,8) | 7.86 (6,9.79) | -1.2(-1.45,-0.95) |
| Argentina | 2395 (1825,3033) | 8.36 (6.39,10.59) | 2401 (1798,2988) | 4.04 (3.03,5.03) | -1.98(-2.17,-1.79) |
| Armenia | 177 (132,228) | 8.02 (5.95,10.42) | 346 (261,441) | 7.96 (6.04,10.12) | -1(-1.4,-0.6) |
| Australia | 1205 (896,1518) | 6.62 (4.88,8.42) | 1434 (1039,1816) | 2.54 (1.84,3.22) | -3.3(-3.41,-3.19) |
| Austria | 783 (574,1039) | 6.08 (4.5,8.01) | 422 (302,542) | 1.77 (1.27,2.25) | -4.47(-4.79,-4.15) |
| Azerbaijan | 187 (128,255) | 4.7 (3.23,6.4) | 487 (332,665) | 6.65 (4.6,9.08) | 1.78(1.47,2.09) |
| Bahamas | 10 (7,12) | 7.58 (5.8,9.62) | 20 (15,26) | 6.2 (4.56,8.13) | -0.64(-0.81,-0.48) |
| Bahrain | 17 (13,21) | 20.33 (15.81,25.8) | 63 (48,79) | 16.94 (12.94,21.19) | -0.97(-1.43,-0.51) |
| Bangladesh | 4142 (2926,5621) | 11.59 (8.3,15.68) | 15480 (10362,21328) | 14.31 (9.61,19.68) | 0.57(0.17,0.97) |
| Barbados | 54 (42,66) | 17.14 (13.4,20.79) | 59 (43,76) | 11.34 (8.33,14.51) | -1.55(-1.77,-1.34) |
| Belarus | 1065 (797,1367) | 8.59 (6.45,11.02) | 1386 (988,1864) | 8.34 (5.93,11.21) | -0.66(-1.06,-0.27) |
| Belgium | 1219 (913,1538) | 7.54 (5.64,9.52) | 832 (583,1056) | 2.63 (1.87,3.31) | -3.3(-3.43,-3.16) |
| Belize | 4 (3,5) | 4.5 (3.45,5.69) | 12 (8,15) | 4.89 (3.55,6.23) | -0.11(-0.7,0.49) |
| Benin | 147 (104,200) | 9.2 (6.59,12.49) | 413 (281,557) | 11.35 (7.82,15.36) | 0.76(0.69,0.82) |
| Bermuda | 5 (4,6) | 8.84 (6.87,11.17) | 7 (5,9) | 4.36 (3.24,5.68) | -2.39(-2.61,-2.18) |
| Bhutan | 9 (6,14) | 6.29 (3.74,9.45) | 37 (26,50) | 6.95 (4.96,9.45) | 0.34(0.3,0.37) |
| Bolivia (Plurinational State of) | 154 (98,212) | 6.42 (4.11,8.82) | 396 (260,569) | 5.6 (3.76,7.92) | -0.39(-0.47,-0.3) |
| Bosnia and Herzegovina | 588 (448,747) | 19.24 (14.49,24.63) | 1349 (985,1759) | 20.69 (15.07,26.91) | -0.03(-0.16,0.1) |
| Botswana | 36 (23,48) | 10.9 (7.11,14.79) | 89 (64,124) | 9.33 (6.62,12.8) | -0.34(-0.6,-0.09) |
| Brazil | 8797 (6831,11031) | 13.14 (10.04,16.58) | 13930 (10614,17222) | 5.9 (4.48,7.31) | -2.28(-2.42,-2.14) |
| Brunei Darussalam | 12 (9,15) | 16.58 (12.62,21.37) | 17 (13,22) | 9.66 (7.3,12.46) | -1.39(-1.7,-1.08) |
| Bulgaria | 2695 (2081,3342) | 30.33 (23.08,37.67) | 4622 (3495,5734) | 30.66 (23.32,38.11) | 0.27(0.05,0.5) |
| Burkina Faso | 146 (100,203) | 5.38 (3.71,7.43) | 448 (307,635) | 6.98 (4.91,9.64) | 1.12(0.98,1.27) |
| Burundi | 141 (91,205) | 8.49 (5.65,12.1) | 186 (120,266) | 6.43 (4.27,9.14) | -1.42(-1.65,-1.18) |
| Cabo Verde | 18 (13,24) | 7.4 (5.31,9.8) | 53 (37,70) | 13.1 (9.37,17.46) | 1.61(1.27,1.94) |
| Cambodia | 218 (155,295) | 8.1 (5.71,10.92) | 880 (622,1211) | 11.47 (8.06,15.71) | 0.97(0.88,1.07) |
| Cameroon | 244 (170,342) | 8.48 (6.03,11.6) | 1064 (720,1577) | 12.96 (8.95,18.77) | 1.55(1.17,1.94) |
| Canada | 1197 (863,1567) | 3.78 (2.73,4.98) | 1798 (1277,2304) | 2.05 (1.46,2.62) | -2.4(-2.61,-2.2) |
| Central African Republic | 75 (50,105) | 12.28 (8.3,17.09) | 146 (94,209) | 13.28 (8.65,19.09) | 0.18(0.1,0.25) |
| Chad | 207 (140,296) | 9.23 (6.25,13.1) | 528 (355,735) | 13.47 (9.05,18.67) | 1.13(0.91,1.35) |
| Chile | 695 (535,864) | 8.13 (6.24,10.17) | 1219 (914,1525) | 4.57 (3.43,5.71) | -1.36(-1.6,-1.12) |
| China | 64571 (49225,85851) | 11.49 (8.72,15.18) | 200876 (148063,260585) | 10.91 (8.03,14.2) | 0.02(-0.3,0.34) |
| Colombia | 804 (618,1021) | 5.82 (4.43,7.38) | 1510 (1099,1941) | 2.69 (1.96,3.47) | -3.15(-3.41,-2.89) |
| Comoros | 9 (6,12) | 7.29 (5.01,10.07) | 21 (14,30) | 6.06 (4.09,8.37) | -0.9(-1.08,-0.71) |
| Congo | 72 (50,98) | 11.41 (8.19,15.33) | 182 (127,244) | 11.68 (8.29,15.53) | -0.14(-0.24,-0.04) |
| Cook Islands | 1 (1,1) | 11.47 (8.68,14.54) | 2 (1,3) | 7.69 (5.52,9.98) | -1.44(-1.61,-1.28) |
| Costa Rica | 69 (54,87) | 4.43 (3.43,5.56) | 187 (138,239) | 3.33 (2.48,4.28) | -1.53(-1.92,-1.15) |
| Croatia | 1184 (917,1467) | 23.8 (18.35,29.38) | 1134 (869,1413) | 10.84 (8.31,13.53) | -2.67(-2.84,-2.51) |
| Cuba | 742 (575,928) | 7.95 (6.17,9.91) | 1588 (1189,1998) | 7.4 (5.54,9.33) | -0.23(-0.37,-0.1) |
| Cyprus | 108 (79,139) | 23.88 (17.57,30.54) | 100 (72,130) | 6.17 (4.44,8.1) | -4.7(-5.04,-4.36) |
| Czechia | 3628 (2839,4499) | 26.92 (21,33.54) | 1801 (1367,2298) | 7.49 (5.7,9.55) | -4.59(-5.07,-4.12) |
| Côte d'Ivoire | 230 (159,306) | 10.38 (7.31,13.56) | 889 (615,1231) | 12.57 (8.77,17.09) | 0.62(0.42,0.83) |
| Democratic People's Republic of Korea | 1089 (746,1509) | 9.65 (6.66,13.36) | 3240 (2246,4397) | 11.05 (7.65,15.12) | 0.37(0.19,0.56) |
| Democratic Republic of the Congo | 963 (633,1384) | 11.3 (7.54,16.07) | 2347 (1478,3498) | 11.14 (7.14,16.41) | -0.22(-0.31,-0.13) |
| Denmark | 402 (294,529) | 4.39 (3.23,5.78) | 387 (284,499) | 2.71 (1.98,3.48) | -1.96(-2.21,-1.71) |
| Djibouti | 4 (2,5) | 5.56 (3.63,8.26) | 24 (17,36) | 7.25 (5.09,10.48) | 0.79(0.75,0.82) |
| Dominica | 7 (6,9) | 13 (10.01,16.53) | 9 (7,12) | 12.87 (9.71,16.55) | -0.06(-0.14,0.02) |
| Dominican Republic | 131 (95,174) | 4.88 (3.53,6.45) | 534 (374,729) | 5.65 (3.96,7.69) | 1.11(0.82,1.41) |
| Ecuador | 243 (189,306) | 5.55 (4.32,6.97) | 614 (450,801) | 4.21 (3.09,5.48) | -0.7(-0.98,-0.42) |
| Egypt | 3294 (2215,4767) | 20.86 (14.26,29.53) | 11224 (8045,15280) | 29.32 (21.39,38.19) | 1.8(1.55,2.05) |
| El Salvador | 89 (66,117) | 3.27 (2.42,4.27) | 207 (148,276) | 3.03 (2.18,4.06) | -0.55(-0.79,-0.31) |
| Equatorial Guinea | 13 (9,19) | 11.21 (7.75,15.86) | 43 (27,64) | 13.46 (8.64,19.61) | 0.49(0.27,0.72) |
| Eritrea | 22 (13,35) | 4.97 (3.1,7.62) | 92 (60,132) | 6.37 (4.19,8.91) | 0.79(0.74,0.84) |
| Estonia | 281 (213,360) | 14.14 (10.68,18.24) | 145 (108,184) | 4.29 (3.19,5.47) | -5.27(-5.87,-4.66) |
| Eswatini | 18 (13,25) | 10.39 (7.45,14.11) | 48 (31,69) | 14.13 (9.52,19.93) | 1.52(1.08,1.95) |
| Ethiopia | 449 (270,743) | 4.13 (2.59,6.59) | 1069 (752,1508) | 3.47 (2.43,4.93) | -0.83(-0.94,-0.72) |
| Fiji | 24 (18,31) | 11.36 (8.54,14.66) | 64 (46,86) | 13.47 (9.56,17.99) | 0.11(-0.15,0.37) |
| Finland | 775 (588,963) | 10.65 (7.99,13.24) | 677 (496,865) | 3.96 (2.91,5.05) | -3.12(-3.28,-2.96) |
| France | 4559 (3406,5847) | 4.89 (3.64,6.24) | 3923 (2789,5071) | 1.88 (1.35,2.42) | -3.05(-3.18,-2.92) |
| Gabon | 48 (34,64) | 10.93 (7.96,14.45) | 88 (63,120) | 12.9 (9.19,17.37) | 0.4(0.23,0.57) |
| Gambia | 22 (15,31) | 9.39 (6.36,13.21) | 108 (70,152) | 14.77 (9.72,20.76) | 1.55(1.48,1.63) |
| Georgia | 390 (278,517) | 7.02 (5.04,9.28) | 970 (726,1232) | 14.55 (10.92,18.56) | 2.83(2.18,3.47) |
| Germany | 14133 (10552,17901) | 10.28 (7.69,13) | 8565 (6311,10893) | 3.33 (2.46,4.22) | -3.71(-3.93,-3.48) |
| Ghana | 518 (374,709) | 13.4 (9.77,18.01) | 2271 (1569,3154) | 20.53 (14.28,28.27) | 1.75(1.47,2.03) |
| Greece | 2242 (1685,2816) | 15.83 (11.83,19.81) | 1997 (1484,2508) | 5.4 (4.03,6.75) | -4.32(-4.68,-3.97) |
| Greenland | 1 (1,2) | 6.72 (4.78,9.19) | 2 (1,3) | 4.83 (3.48,6.65) | -1.32(-1.57,-1.07) |
| Grenada | 14 (11,18) | 16.7 (12.85,21.04) | 10 (7,12) | 11.07 (8.41,13.87) | -1.32(-1.55,-1.08) |
| Guam | 4 (3,5) | 9.51 (7.15,12.03) | 7 (5,8) | 3.05 (2.24,3.88) | -3.15(-3.6,-2.7) |
| Guatemala | 91 (68,116) | 4.32 (3.22,5.51) | 326 (247,414) | 3.66 (2.78,4.65) | -1.15(-1.55,-0.76) |
| Guinea | 208 (138,284) | 8.08 (5.34,10.88) | 518 (355,723) | 12.12 (8.47,16.71) | 1.41(1.22,1.6) |
| Guinea-Bissau | 35 (24,49) | 13.15 (9.13,17.77) | 72 (50,100) | 17.03 (11.8,23) | 1.06(0.96,1.16) |
| Guyana | 77 (60,95) | 24.88 (19.55,30.97) | 96 (70,124) | 19.76 (14.46,25.38) | -0.22(-0.45,0.01) |
| Haiti | 390 (270,519) | 19.39 (13.59,25.85) | 739 (495,1068) | 16.47 (11.14,23.41) | -0.37(-0.45,-0.3) |
| Honduras | 94 (66,130) | 6.06 (4.32,8.26) | 494 (344,671) | 10.43 (7.32,14.12) | 1.82(1.53,2.12) |
| Hungary | 2939 (2260,3620) | 21.46 (16.54,26.5) | 1897 (1442,2399) | 8.58 (6.53,10.82) | -3.43(-3.67,-3.19) |
| Iceland | 17 (12,21) | 5.25 (3.89,6.69) | 18 (13,23) | 2.46 (1.77,3.15) | -2.53(-2.73,-2.33) |
| India | 19175 (14238,26176) | 6.21 (4.64,8.41) | 63698 (48247,84356) | 6.64 (5.03,8.72) | 0.08(-0.07,0.23) |
| Indonesia | 5527 (3913,7376) | 8.99 (6.38,11.93) | 24150 (16161,33081) | 16.43 (11.42,22.16) | 2.13(1.89,2.38) |
| Iran (Islamic Republic of) | 2231 (1700,2867) | 13.94 (10.56,18.06) | 6880 (5283,8634) | 10.81 (8.23,13.58) | -0.9(-1.04,-0.77) |
| Iraq | 1896 (1473,2425) | 26.93 (20.96,34.28) | 5733 (4099,7546) | 34.8 (25.35,45.14) | 0.32(0.1,0.54) |
| Ireland | 333 (252,416) | 8.59 (6.49,10.78) | 225 (163,283) | 2.56 (1.85,3.21) | -3.85(-4.08,-3.63) |
| Israel | 261 (196,325) | 5.84 (4.43,7.36) | 341 (248,426) | 2.33 (1.71,2.9) | -3.54(-3.78,-3.3) |
| Italy | 9417 (7213,12019) | 10.73 (8.16,13.61) | 8056 (5801,10337) | 3.7 (2.69,4.72) | -3.7(-3.89,-3.51) |
| Jamaica | 163 (125,208) | 8.58 (6.59,10.97) | 245 (167,322) | 7.25 (4.92,9.59) | -0.37(-0.74,0) |
| Japan | 17218 (13340,21253) | 11.63 (8.93,14.39) | 19282 (13376,24645) | 3.08 (2.22,3.88) | -4.58(-4.74,-4.42) |
| Jordan | 220 (162,288) | 24.52 (18.21,31.82) | 748 (561,994) | 15.62 (11.6,20.77) | -1.89(-2.23,-1.55) |
| Kazakhstan | 1416 (1055,1858) | 13.63 (10.08,17.89) | 2279 (1688,2919) | 17.67 (13.02,22.52) | 0.54(0.08,1) |
| Kenya | 224 (152,316) | 4.04 (2.72,5.7) | 730 (512,1027) | 5.26 (3.58,7.47) | 1.05(0.96,1.14) |
| Kiribati | 3 (2,4) | 12.49 (8.85,16.42) | 7 (5,9) | 15.97 (12.23,20.56) | 0.83(0.77,0.89) |
| Kuwait | 28 (22,36) | 7.38 (5.64,9.38) | 145 (109,186) | 7.24 (5.39,9.37) | 0.33(-0.79,1.45) |
| Kyrgyzstan | 246 (181,326) | 9.62 (7.07,12.86) | 295 (218,387) | 7.62 (5.65,10.09) | -1.29(-1.67,-0.91) |
| Lao People's Democratic Republic | 225 (161,309) | 16.93 (12.24,22.44) | 428 (295,581) | 13.94 (9.82,18.68) | -0.85(-0.94,-0.77) |
| Latvia | 537 (403,689) | 15.31 (11.5,19.77) | 681 (513,865) | 13.82 (10.43,17.55) | -0.71(-0.97,-0.46) |
| Lebanon | 236 (173,316) | 14.3 (10.52,19.16) | 482 (365,627) | 7.13 (5.41,9.26) | -2.42(-2.69,-2.16) |
| Lesotho | 35 (24,50) | 5.63 (3.84,7.93) | 98 (65,145) | 13.41 (9.05,18.84) | 3.9(3.33,4.47) |
| Liberia | 93 (66,127) | 10.94 (7.91,14.67) | 184 (127,261) | 13.28 (9.24,18.75) | 0.63(0.53,0.73) |
| Libya | 125 (86,177) | 8.08 (5.55,11.48) | 465 (314,668) | 11.63 (7.78,16.69) | 1.85(1.58,2.11) |
| Lithuania | 335 (248,424) | 7.46 (5.55,9.46) | 551 (416,707) | 7.78 (5.87,9.93) | -0.06(-0.43,0.31) |
| Luxembourg | 60 (45,77) | 11.17 (8.39,14.39) | 42 (31,53) | 3.25 (2.44,4.11) | -3.67(-3.85,-3.48) |
| Madagascar | 253 (183,344) | 7.65 (5.54,10.19) | 507 (315,704) | 8.65 (5.57,11.92) | 0.3(0.22,0.38) |
| Malawi | 118 (83,160) | 5.42 (3.81,7.37) | 361 (246,492) | 7.67 (5.17,10.49) | 0.92(0.77,1.07) |
| Malaysia | 832 (623,1049) | 10.96 (8.2,13.84) | 2295 (1726,2875) | 10.39 (7.78,13.04) | 0.02(-0.14,0.17) |
| Maldives | 6 (5,8) | 12.97 (9.78,16.82) | 19 (14,25) | 7.75 (5.71,10.07) | -2(-2.11,-1.9) |
| Mali | 205 (137,297) | 8 (5.44,11.28) | 570 (385,809) | 9.54 (6.48,13.47) | 0.78(0.67,0.9) |
| Malta | 34 (26,42) | 8.59 (6.47,10.87) | 35 (26,44) | 2.95 (2.19,3.73) | -3.53(-3.78,-3.29) |
| Marshall Islands | 2 (2,3) | 20.36 (14.7,26.62) | 3 (2,5) | 20.17 (14.51,26.84) | -0.1(-0.13,-0.06) |
| Mauritania | 72 (48,100) | 9.59 (6.52,13.3) | 169 (112,253) | 10.32 (6.9,15.3) | 0(-0.22,0.22) |
| Mauritius | 116 (92,143) | 20.7 (16.25,25.54) | 143 (113,182) | 8.73 (6.84,11.09) | -3.99(-4.51,-3.48) |
| Mexico | 2896 (2276,3584) | 9.17 (7.17,11.37) | 4440 (3421,5615) | 3.99 (3.07,5.05) | -2.77(-2.92,-2.63) |
| Micronesia (Federated States of) | 5 (4,7) | 13.53 (9.7,18.13) | 6 (4,8) | 12.07 (8.49,16.2) | -0.48(-0.52,-0.44) |
| Monaco | 10 (7,13) | 11.02 (7.59,15.01) | 8 (6,11) | 5.86 (4.23,8.02) | -2.2(-2.41,-1.99) |
| Mongolia | 14 (9,20) | 1.65 (1.06,2.35) | 43 (29,60) | 2.76 (1.82,3.89) | 1.67(1.33,2.02) |
| Montenegro | 41 (31,54) | 7.43 (5.57,9.78) | 125 (90,170) | 15.3 (11.1,20.66) | 2.79(2.52,3.07) |
| Morocco | 2141 (1499,3017) | 18.13 (12.7,25.3) | 6976 (5012,9265) | 25.22 (18.1,33.4) | 1.3(1.22,1.39) |
| Mozambique | 250 (176,334) | 6.81 (4.75,9.06) | 771 (504,1068) | 10.57 (7.24,14.55) | 1.94(1.77,2.11) |
| Myanmar | 2628 (1843,3627) | 16.64 (11.81,22.37) | 5317 (3711,7276) | 14.5 (10.13,19.77) | -0.66(-0.75,-0.57) |
| Namibia | 48 (35,62) | 12.3 (9,16.16) | 125 (89,169) | 13.48 (9.53,18.12) | 0.17(-0.09,0.42) |
| Nauru | 1 (0,1) | 19.14 (13.24,25.25) | 1 (1,1) | 19.49 (14.23,26.15) | -0.14(-0.34,0.07) |
| Nepal | 468 (311,668) | 7.77 (5.25,11.05) | 1504 (1012,2081) | 8.51 (5.86,11.78) | 0.23(0.13,0.33) |
| Netherlands | 1301 (963,1649) | 6.21 (4.6,7.84) | 1343 (980,1727) | 3.23 (2.36,4.15) | -2.71(-3.02,-2.41) |
| New Zealand | 272 (208,343) | 7.14 (5.44,9.12) | 370 (268,471) | 3.82 (2.78,4.87) | -2.2(-2.36,-2.03) |
| Nicaragua | 54 (40,70) | 4.47 (3.32,5.79) | 126 (92,167) | 3.13 (2.28,4.16) | -1.05(-1.25,-0.84) |
| Niger | 117 (73,181) | 7.26 (4.62,10.81) | 471 (297,707) | 9.35 (6.11,13.72) | 0.98(0.93,1.04) |
| Nigeria | 2803 (1842,3900) | 8.76 (5.82,12.06) | 5367 (3774,7368) | 8.8 (6.24,12.04) | -0.09(-0.18,0) |
| Niue | 0 (0,0) | 13.59 (10.29,17.87) | 0 (0,0) | 14.5 (10.9,18.91) | 0(-0.07,0.07) |
| North Macedonia | 532 (403,660) | 35.13 (26.63,43.66) | 1149 (832,1441) | 50.36 (36.42,63.75) | 0.81(0.24,1.38) |
| Northern Mariana Islands | 1 (1,1) | 11.99 (9.06,15.7) | 3 (2,3) | 9.04 (6.86,11.53) | -1.47(-1.75,-1.19) |
| Norway | 815 (621,1020) | 10.21 (7.78,12.75) | 366 (266,464) | 2.84 (2.1,3.63) | -4.35(-4.49,-4.22) |
| Oman | 65 (46,90) | 12.98 (9.18,17.74) | 159 (117,208) | 13.53 (10.02,17.64) | 0.7(0.34,1.06) |
| Pakistan | 3371 (2276,4731) | 7.59 (5.15,10.73) | 9133 (6631,12530) | 10.71 (7.81,14.65) | 1.01(0.82,1.21) |
| Palau | 1 (1,2) | 16.96 (12.97,22.05) | 2 (2,3) | 17.43 (13.06,22.97) | 0.26(0.17,0.34) |
| Palestine | 169 (120,220) | 25.29 (17.87,32.71) | 307 (225,397) | 19.14 (14.16,24.67) | -0.95(-1.27,-0.63) |
| Panama | 74 (55,93) | 5.65 (4.26,7.19) | 214 (151,280) | 4.62 (3.24,6.05) | -0.89(-1.11,-0.67) |
| Papua New Guinea | 75 (49,111) | 8 (5.36,11.53) | 231 (153,339) | 8.39 (5.71,12.17) | 0.1(-0.01,0.22) |
| Paraguay | 152 (113,196) | 8 (5.92,10.3) | 392 (278,520) | 7.66 (5.42,10.17) | 0.08(-0.11,0.28) |
| Peru | 271 (196,360) | 2.66 (1.92,3.53) | 765 (527,1030) | 2.33 (1.6,3.15) | -0.89(-1.29,-0.5) |
| Philippines | 1424 (1067,1820) | 8.17 (6.06,10.41) | 4265 (3178,5468) | 7.16 (5.28,9.17) | -0.18(-0.28,-0.08) |
| Poland | 9003 (7071,11129) | 22.81 (17.84,28.24) | 8109 (6298,10073) | 10.11 (7.86,12.55) | -3.02(-3.14,-2.91) |
| Portugal | 3502 (2713,4359) | 28.58 (21.96,35.8) | 2170 (1620,2707) | 6.34 (4.78,7.9) | -5.49(-5.75,-5.24) |
| Puerto Rico | 222 (174,273) | 6.89 (5.38,8.45) | 245 (176,309) | 2.55 (1.88,3.19) | -3.58(-3.82,-3.33) |
| Qatar | 8 (6,11) | 19.57 (14.38,25.42) | 37 (27,49) | 12.2 (8.97,15.88) | -2.05(-2.85,-1.25) |
| Republic of Korea | 3523 (2652,4487) | 18.86 (14.09,24.14) | 4744 (3437,6099) | 5.2 (3.74,6.72) | -4.79(-5.03,-4.55) |
| Republic of Moldova | 321 (249,420) | 10.07 (7.77,13.09) | 502 (380,642) | 8.23 (6.23,10.52) | -0.26(-0.75,0.23) |
| Romania | 4750 (3555,6049) | 22.14 (16.96,27.93) | 7808 (5951,9887) | 18.21 (13.92,22.96) | -1.03(-1.28,-0.77) |
| Russian Federation | 28626 (21955,36920) | 18.89 (14.42,24.33) | 37442 (28602,47332) | 15.36 (11.73,19.51) | -1.37(-1.87,-0.87) |
| Rwanda | 121 (84,176) | 7.68 (5.38,10.86) | 198 (124,289) | 5.25 (3.3,7.53) | -2.02(-2.41,-1.64) |
| Saint Kitts and Nevis | 9 (7,12) | 26.2 (20.06,32.86) | 7 (6,9) | 15.98 (12.36,20.02) | -1.41(-1.6,-1.22) |
| Saint Lucia | 16 (13,20) | 24.34 (18.74,30.6) | 26 (20,34) | 11.68 (8.69,14.95) | -3.24(-3.69,-2.78) |
| Saint Vincent and the Grenadines | 9 (7,12) | 15.22 (11.73,19.08) | 13 (10,16) | 10.65 (8.05,13.44) | -0.96(-1.24,-0.68) |
| Samoa | 7 (5,9) | 11.01 (7.96,14.21) | 13 (9,17) | 11.66 (8.52,15.28) | 0.23(0.12,0.34) |
| San Marino | 3 (2,3) | 6.9 (4.89,9.07) | 3 (2,5) | 2.78 (1.81,4.04) | -2.37(-2.74,-2) |
| Sao Tome and Principe | 4 (3,5) | 7.32 (5.27,9.68) | 10 (7,13) | 12.32 (8.99,16.8) | 1.98(1.88,2.08) |
| Saudi Arabia | 716 (505,933) | 17.52 (12.41,22.76) | 1674 (1230,2237) | 16.15 (12.04,21.59) | -0.65(-0.82,-0.49) |
| Senegal | 347 (252,448) | 14.68 (10.66,19) | 935 (651,1311) | 16.54 (11.46,23.02) | 0.33(0.27,0.39) |
| Serbia | 3174 (2439,4055) | 41.71 (32.48,53.41) | 4928 (3615,6429) | 27.85 (20.5,36.32) | -1.96(-2.25,-1.68) |
| Seychelles | 7 (5,9) | 12.32 (9.12,16.02) | 10 (7,13) | 10.38 (7.44,13.31) | -0.3(-0.51,-0.09) |
| Sierra Leone | 166 (119,223) | 10.14 (7.28,13.55) | 345 (235,477) | 12.55 (8.54,17.07) | 0.95(0.78,1.13) |
| Singapore | 212 (169,262) | 12.44 (9.78,15.34) | 129 (96,161) | 1.59 (1.18,1.99) | -6.49(-6.98,-6) |
| Slovakia | 896 (679,1153) | 15.72 (11.75,20.24) | 999 (739,1290) | 10.35 (7.66,13.37) | -1.48(-1.58,-1.38) |
| Slovenia | 361 (278,451) | 14.76 (11.38,18.37) | 322 (244,402) | 5.65 (4.28,7.06) | -3.11(-3.29,-2.93) |
| Solomon Islands | 6 (4,9) | 8.55 (5.92,11.9) | 19 (12,28) | 9.18 (6.19,13.31) | 0.15(0.11,0.18) |
| Somalia | 62 (37,96) | 5.27 (3.29,7.91) | 163 (94,261) | 5.36 (3.16,8.32) | 0.14(0.09,0.18) |
| South Africa | 982 (675,1285) | 6.09 (4.2,7.95) | 3527 (2696,4431) | 10.25 (7.84,12.92) | 1.96(1.41,2.5) |
| South Sudan | 97 (64,141) | 5.44 (3.62,7.81) | 132 (86,195) | 5.74 (3.83,8.32) | 0.03(-0.13,0.19) |
| Spain | 6540 (4882,8146) | 12.28 (9.19,15.33) | 4048 (2904,5086) | 2.82 (2.06,3.53) | -4.78(-4.93,-4.64) |
| Sri Lanka | 1056 (791,1355) | 15.36 (11.63,19.77) | 2933 (1954,4008) | 13.56 (9.11,18.58) | 0.43(0.09,0.76) |
| Sudan | 1128 (740,1599) | 16.07 (10.54,22.46) | 2412 (1573,3377) | 16.71 (10.96,23.3) | -0.03(-0.09,0.02) |
| Suriname | 24 (18,29) | 10.92 (8.07,13.66) | 53 (36,71) | 9.25 (6.41,12.46) | -0.43(-0.69,-0.17) |
| Sweden | 1114 (835,1419) | 6.29 (4.71,8.03) | 811 (585,1048) | 2.78 (2.01,3.6) | -2.77(-3.07,-2.48) |
| Switzerland | 826 (616,1061) | 6.88 (5.13,8.81) | 567 (395,730) | 2.22 (1.57,2.86) | -3.55(-3.67,-3.44) |
| Syrian Arab Republic | 531 (378,714) | 13.57 (9.75,18.2) | 1362 (969,1870) | 14.98 (10.88,20.35) | -0.19(-0.41,0.03) |
| Taiwan (Province of China) | 1082 (838,1340) | 9.52 (7.34,11.92) | 1302 (982,1624) | 2.91 (2.21,3.63) | -3.9(-4.12,-3.68) |
| Tajikistan | 224 (156,303) | 9.54 (6.62,12.83) | 453 (331,603) | 11.94 (8.79,15.74) | 0.51(0.09,0.93) |
| Thailand | 1697 (1227,2210) | 6.73 (4.81,8.75) | 5260 (3637,7068) | 4.85 (3.35,6.5) | -1.7(-1.95,-1.45) |
| Timor-Leste | 10 (7,13) | 6.8 (4.82,9.15) | 66 (42,97) | 10.63 (6.88,15.3) | 1.44(1.37,1.51) |
| Togo | 72 (50,97) | 9.05 (6.5,12.17) | 267 (176,379) | 11.5 (7.74,15.96) | 0.69(0.54,0.84) |
| Tokelau | 0 (0,0) | 16.85 (12.49,21.99) | 0 (0,0) | 14.61 (10.52,18.74) | -0.53(-0.58,-0.49) |
| Tonga | 3 (2,3) | 6.4 (4.58,8.29) | 5 (4,7) | 7.09 (5.08,9.39) | 0.5(0.37,0.62) |
| Trinidad and Tobago | 111 (87,139) | 16.88 (13.12,21.03) | 170 (122,228) | 9.38 (6.76,12.62) | -2.05(-2.31,-1.8) |
| Tunisia | 440 (316,592) | 12.57 (9.23,16.92) | 1431 (914,1978) | 12.61 (8.02,17.4) | -0.21(-0.37,-0.05) |
| Turkmenistan | 111 (80,145) | 7.16 (5.14,9.36) | 451 (318,636) | 13.89 (9.93,19.33) | 1.84(1.38,2.3) |
| Tuvalu | 0 (0,1) | 11.71 (8.43,15.21) | 1 (1,1) | 10.07 (7.32,13.58) | -0.57(-0.63,-0.52) |
| Turkey | 3193 (2385,4122) | 12.3 (9.18,15.85) | 8372 (6098,11047) | 10.48 (7.66,13.92) | -0.22(-0.61,0.18) |
| Uganda | 238 (159,338) | 5.72 (3.83,8.12) | 497 (336,698) | 5.21 (3.57,7.32) | -0.88(-1.13,-0.63) |
| Ukraine | 10506 (8089,13475) | 16.38 (12.39,21.08) | 8939 (6393,12322) | 11.02 (7.92,15.19) | -1.98(-2.29,-1.65) |
| United Arab Emirates | 44 (31,59) | 18.51 (13.22,24.86) | 179 (127,230) | 18.47 (13.6,23.65) | 2.19(1.48,2.92) |
| United Kingdom | 6955 (5313,8920) | 7.04 (5.35,9.03) | 3989 (2941,5038) | 2.42 (1.79,3.06) | -3.68(-3.94,-3.42) |
| United Republic of Tanzania | 254 (172,368) | 3.95 (2.7,5.77) | 1202 (790,1692) | 6.8 (4.54,9.43) | 1.74(1.55,1.93) |
| United States of America | 17064 (12931,21300) | 4.97 (3.75,6.22) | 28920 (21181,35566) | 4.36 (3.21,5.33) | -0.91(-1.21,-0.61) |
| United States Virgin Islands | 5 (4,6) | 8.91 (6.67,11.39) | 9 (6,12) | 4.91 (3.58,6.65) | -1.69(-1.84,-1.55) |
| Uruguay | 325 (250,414) | 8.34 (6.42,10.65) | 396 (294,501) | 5.7 (4.25,7.18) | -1.35(-1.56,-1.14) |
| Uzbekistan | 534 (387,691) | 5.12 (3.66,6.72) | 1878 (1385,2470) | 9.76 (7.23,12.81) | 1.61(1.26,1.97) |
| Vanuatu | 5 (3,6) | 12.74 (8.83,17.21) | 13 (9,17) | 12.02 (8.51,16.24) | -0.34(-0.41,-0.28) |
| Venezuela (Bolivarian Republic of) | 382 (290,484) | 4.95 (3.75,6.27) | 1387 (968,1891) | 5.3 (3.7,7.23) | -0.04(-0.31,0.23) |
| Viet Nam | 3805 (2700,5131) | 11.71 (8.31,15.91) | 11166 (7591,14881) | 14.68 (10.09,19.46) | 0.97(0.8,1.14) |
| Yemen | 499 (329,715) | 15.36 (10.25,21.69) | 1904 (1234,2783) | 19.62 (12.78,28.46) | 0.73(0.69,0.77) |
| Zambia | 138 (93,196) | 8.04 (5.46,11.33) | 462 (320,655) | 11.08 (7.75,15.57) | 1(0.89,1.1) |
| Zimbabwe | 182 (133,235) | 7.39 (5.38,9.58) | 460 (334,625) | 11.11 (8.06,14.89) | 2.03(1.52,2.53) |
