# Supplementary material for: The persistent challenge of ischemic stroke burden from high fasting plasma glucose: a global perspective
Source: Front Endocrinol (Lausanne). 2025 May 6;16:1490428. doi: 10.3389/fendo.2025.1490428 (PMC12088946; doi:10.3389/fendo.2025.1490428)
Supplement: Supplementary file 4 [file Table2.docx]

Table S2 DALYs and ASDR of ischemic stroke attributable to HFPG in 204 countries and territories in 1990 and 2021, and the temporal trends from 1990 to 2021.

| **DALY** | 1990 | | 2021 | | 1990–2021 |
| --- | --- | --- | --- | --- | --- |
| Location | DALYs cases  No. (95% UI) | ASDR per 100,000  No. (95% UI) | DALYs cases  No. (95% UI) | ASDR per 100,000  No. (95% UI) | EAPC in ASDR  No. (95% CI) |
| Afghanistan | 22870 (15332,33061) | 368.95 (253.55,527.12) | 38326 (25339,53934) | 479.58 (325.41,674.55) | 0.78(0.69,0.87) |
| Albania | 1843 (1303,2448) | 109.36 (77.91,145.3) | 5119 (3535,7083) | 119.2 (82.18,165.58) | 0.69(0.47,0.91) |
| Algeria | 27843 (20014,37428) | 323.08 (234.45,425.17) | 86792 (62559,117251) | 307.26 (220.58,408.86) | -0.08(-0.12,-0.04) |
| American Samoa | 39 (30,48) | 222.08 (169.86,276.23) | 89 (68,113) | 213.78 (163.84,269.62) | -0.39(-0.53,-0.25) |
| Andorra | 32 (22,43) | 62.46 (43.29,85.43) | 71 (49,96) | 42.15 (29.16,57.07) | -1.13(-1.35,-0.91) |
| Angola | 4239 (2996,5912) | 159.61 (115.41,220.9) | 14762 (10277,19834) | 181.09 (127.72,242.25) | 0.2(0.11,0.28) |
| Antigua and Barbuda | 98 (73,123) | 166.5 (124.49,207.79) | 113 (88,140) | 120.83 (93.11,149.38) | -1.27(-1.5,-1.04) |
| Argentina | 41954 (31817,52963) | 137.14 (103.39,173.25) | 43550 (32987,53519) | 74.42 (56.41,91.45) | -1.74(-1.89,-1.58) |
| Armenia | 3507 (2574,4527) | 146.63 (107.99,190.4) | 6382 (4813,8114) | 145.89 (111.08,184.82) | -0.93(-1.28,-0.58) |
| Australia | 19589 (14846,24728) | 102.01 (76.78,130.31) | 23320 (17460,29263) | 45.19 (34.1,56.84) | -2.84(-2.94,-2.74) |
| Austria | 11465 (8478,15142) | 88.11 (65.86,116.21) | 7985 (5877,10337) | 37.32 (27.3,47.9) | -3.04(-3.29,-2.79) |
| Azerbaijan | 4060 (2843,5498) | 91.46 (64.24,123.8) | 10882 (7761,14750) | 127.96 (91.14,171.29) | 1.46(1.21,1.72) |
| Bahamas | 173 (132,221) | 126.31 (97.15,160.31) | 361 (265,471) | 101.96 (75.08,132.89) | -0.64(-0.76,-0.51) |
| Bahrain | 365 (281,456) | 323.51 (252.14,408.25) | 1389 (1054,1735) | 259.11 (198.53,318.14) | -1.19(-1.59,-0.79) |
| Bangladesh | 73043 (51732,100008) | 185.48 (132.69,250.96) | 263546 (176806,365612) | 219.45 (146.84,301.13) | 0.56(0.28,0.83) |
| Barbados | 792 (620,973) | 247.29 (193.94,303.58) | 899 (669,1150) | 170.32 (127.09,217.57) | -1.43(-1.64,-1.22) |
| Belarus | 20640 (15456,26593) | 160.61 (120.81,208.27) | 25209 (17865,33813) | 152.04 (108.04,203.54) | -0.83(-1.28,-0.39) |
| Belgium | 18017 (13468,22971) | 110.17 (82.06,140.58) | 12901 (9544,16235) | 45.83 (34.4,57.7) | -2.8(-2.98,-2.63) |
| Belize | 65 (50,82) | 72.64 (55.32,91.17) | 200 (147,253) | 78.01 (57.14,98.47) | -0.11(-0.66,0.44) |
| Benin | 2632 (1860,3548) | 152.02 (108.69,204.95) | 7903 (5488,10579) | 192.41 (134.24,256.33) | 0.83(0.76,0.9) |
| Bermuda | 79 (61,100) | 137.29 (106.2,173.97) | 107 (80,136) | 70.54 (52.61,90.61) | -2.19(-2.44,-1.94) |
| Bhutan | 196 (127,287) | 108.92 (70.02,161.01) | 655 (463,888) | 118.03 (83.87,160.08) | 0.24(0.21,0.27) |
| Bolivia (Plurinational State of) | 2870 (1850,3989) | 106.85 (69.39,146.26) | 7194 (4750,10427) | 91.27 (60.69,130.09) | -0.49(-0.58,-0.4) |
| Bosnia and Herzegovina | 11217 (8625,14307) | 328.66 (249.68,416.05) | 22646 (16581,29390) | 347.1 (254.83,451.21) | -0.06(-0.17,0.05) |
| Botswana | 729 (488,970) | 182.55 (121.23,243.19) | 1902 (1368,2542) | 171.27 (124.73,230.19) | -0.08(-0.32,0.15) |
| Brazil | 164807 (128139,206691) | 217.42 (168.74,273.59) | 246564 (192306,303909) | 101.58 (79.1,125.36) | -2.26(-2.4,-2.13) |
| Brunei Darussalam | 248 (184,320) | 304.15 (228.74,390.78) | 411 (309,521) | 167.33 (127.24,211.04) | -1.81(-2.03,-1.59) |
| Bulgaria | 50261 (38907,62037) | 468.59 (360.2,576.6) | 74482 (55835,91485) | 488.61 (367.85,601.99) | 0.29(0.1,0.48) |
| Burkina Faso | 2896 (2010,3989) | 88.12 (61.7,121.48) | 8780 (6036,12463) | 118.34 (82.34,165.31) | 1.22(1.1,1.34) |
| Burundi | 2774 (1811,4058) | 145.32 (96.25,207.29) | 3815 (2481,5397) | 110.45 (73.55,155.3) | -1.4(-1.64,-1.16) |
| Cabo Verde | 317 (229,419) | 131.2 (94.91,173.34) | 946 (679,1253) | 229.67 (165.39,302.84) | 1.57(1.29,1.85) |
| Cambodia | 4172 (3031,5526) | 128.9 (93.03,171.16) | 16219 (11501,21874) | 176.9 (125.6,240.73) | 0.85(0.75,0.95) |
| Cameroon | 4819 (3383,6850) | 142.4 (102.03,196.85) | 22238 (15119,32366) | 227.34 (158.11,328.03) | 1.68(1.28,2.08) |
| Canada | 20260 (14897,26077) | 62.1 (45.65,79.86) | 33826 (24888,43401) | 42.14 (31.1,53.9) | -1.56(-1.75,-1.38) |
| Central African Republic | 1732 (1174,2427) | 218.44 (149.68,301.4) | 3439 (2271,4857) | 235.38 (157.5,331.5) | 0.16(0.08,0.24) |
| Chad | 3989 (2762,5660) | 159.35 (110.56,223.87) | 10842 (7345,15082) | 236.86 (162.36,327.79) | 1.17(0.94,1.4) |
| Chile | 12490 (9596,15524) | 136.16 (104.59,169.14) | 20444 (15725,25630) | 77.62 (59.65,97.2) | -1.49(-1.67,-1.31) |
| China | 1391700 (1055720,1844115) | 202.39 (152.94,265.99) | 3924772 (2973434,5032509) | 195.67 (146.01,251.81) | 0.09(-0.16,0.34) |
| Colombia | 15065 (11542,19144) | 99.76 (76.44,126.02) | 26373 (19144,33651) | 47.71 (34.69,60.76) | -3.09(-3.38,-2.79) |
| Comoros | 174 (118,245) | 123.1 (85.1,168.9) | 425 (287,581) | 106.69 (72.26,146.5) | -0.74(-0.91,-0.57) |
| Congo | 1551 (1088,2083) | 197.97 (142.18,261.96) | 3987 (2815,5260) | 206.89 (148.6,272.14) | -0.07(-0.17,0.03) |
| Cook Islands | 23 (18,29) | 215.68 (165.07,267.92) | 43 (32,54) | 165.62 (122.82,209.92) | -0.99(-1.13,-0.84) |
| Costa Rica | 1177 (913,1472) | 72.49 (56.31,90.7) | 3029 (2286,3825) | 55.03 (41.44,69.52) | -1.46(-1.8,-1.12) |
| Croatia | 19407 (14909,24143) | 367.85 (281.57,457.66) | 17383 (13201,21746) | 171.02 (129.9,213.09) | -2.56(-2.71,-2.41) |
| Cuba | 12284 (9441,15252) | 124.26 (95.79,153.97) | 25270 (18761,31719) | 122.64 (91.12,153.54) | 0.01(-0.12,0.13) |
| Cyprus | 1591 (1164,2013) | 296.72 (216.86,378.72) | 1381 (1000,1772) | 75.97 (55.58,98) | -4.76(-5.02,-4.5) |
| Czechia | 59560 (46416,74037) | 425.78 (332.33,527.39) | 31208 (24000,39452) | 132.67 (102.14,167.8) | -4.16(-4.5,-3.81) |
| Côte d'Ivoire | 5144 (3583,6869) | 181.68 (129.32,238.53) | 19270 (13380,27288) | 224.18 (156.23,305.91) | 0.62(0.41,0.84) |
| Democratic People's Republic of Korea | 24743 (16978,34116) | 185.82 (128.7,253.69) | 69336 (48683,92017) | 220.79 (156.17,292.52) | 0.51(0.34,0.68) |
| Democratic Republic of the Congo | 22114 (14933,30879) | 199.4 (137.56,279.53) | 50580 (33214,74321) | 194.63 (128.27,284.96) | -0.25(-0.34,-0.16) |
| Denmark | 6391 (4629,8330) | 71.12 (51.77,92.37) | 6021 (4479,7597) | 44.19 (32.7,55.76) | -1.91(-2.14,-1.68) |
| Djibouti | 78 (51,116) | 95.5 (63.33,139.69) | 549 (388,798) | 129.2 (92.5,185.51) | 0.91(0.88,0.94) |
| Dominica | 115 (88,145) | 193.65 (148.24,242.69) | 152 (117,194) | 196.21 (149.94,248.8) | 0.05(-0.05,0.14) |
| Dominican Republic | 2310 (1700,3046) | 75.12 (54.81,98.41) | 9710 (6888,13341) | 100.58 (71.27,137.94) | 1.45(1.24,1.66) |
| Ecuador | 4509 (3476,5690) | 94.96 (73.21,119.66) | 10660 (7864,14002) | 69.1 (51.08,90.68) | -0.91(-1.17,-0.65) |
| Egypt | 64204 (42321,94886) | 330.66 (224.57,475.49) | 238514 (170868,329341) | 495.62 (362.45,660.66) | 1.86(1.64,2.08) |
| El Salvador | 1629 (1219,2124) | 58.29 (43.56,75.73) | 3467 (2539,4558) | 53.4 (38.89,70.33) | -0.61(-0.85,-0.37) |
| Equatorial Guinea | 286 (196,400) | 198.89 (139.24,275.93) | 926 (594,1336) | 242.03 (158.68,349.11) | 0.56(0.34,0.78) |
| Eritrea | 549 (337,847) | 86.47 (54.05,130.37) | 1984 (1328,2841) | 110.29 (74.07,153.75) | 0.77(0.7,0.84) |
| Estonia | 4910 (3714,6283) | 239.44 (181.16,304.97) | 2398 (1781,3084) | 79.31 (59.09,102.2) | -4.87(-5.4,-4.33) |
| Eswatini | 351 (250,486) | 171.7 (124.54,235.35) | 1018 (668,1444) | 247.08 (166.47,341.37) | 1.62(1.18,2.06) |
| Ethiopia | 10508 (6691,16922) | 75.23 (48.68,116.86) | 20873 (14869,28866) | 60.54 (43.13,83.44) | -1.01(-1.14,-0.88) |
| Fiji | 615 (456,780) | 225.05 (166.66,287.56) | 1568 (1149,2033) | 257.11 (189.12,332.44) | 0.08(-0.11,0.27) |
| Finland | 13043 (10051,16189) | 175.8 (135.34,218.56) | 10869 (8260,13624) | 70.88 (53.74,88.6) | -2.86(-2.98,-2.73) |
| France | 65277 (49278,83431) | 71.1 (54.18,90.85) | 60436 (44679,77637) | 34.56 (25.29,44.36) | -2.28(-2.39,-2.17) |
| Gabon | 954 (686,1252) | 194.7 (142.36,255.12) | 1897 (1351,2550) | 233.61 (169.26,307.51) | 0.47(0.31,0.63) |
| Gambia | 461 (314,653) | 165.11 (113.02,231.2) | 2119 (1393,2990) | 258.41 (171.6,363.78) | 1.5(1.41,1.6) |
| Georgia | 7049 (5136,9418) | 119.8 (86.78,159.38) | 15667 (11754,19990) | 245.21 (182.92,313.14) | 2.67(2.18,3.16) |
| Germany | 216109 (164303,272779) | 156.73 (118.23,197.07) | 148573 (113916,190775) | 64.14 (48.7,81.95) | -2.95(-3.17,-2.73) |
| Ghana | 10693 (7691,14675) | 227.39 (167.39,305.06) | 48354 (33797,66320) | 362.18 (254.71,494.68) | 1.89(1.62,2.16) |
| Greece | 32073 (24435,40405) | 214.01 (162,269) | 25775 (19199,32356) | 78.12 (58.58,98.25) | -4.04(-4.33,-3.75) |
| Greenland | 26 (19,35) | 112.84 (82.23,152.81) | 41 (30,55) | 79.53 (57.7,107.43) | -1.36(-1.54,-1.17) |
| Grenada | 223 (170,281) | 280.2 (213.68,354.89) | 170 (130,214) | 173.76 (132.8,218.5) | -1.53(-1.72,-1.33) |
| Guam | 100 (78,125) | 176.98 (138.05,220.49) | 208 (155,266) | 98.8 (73.23,126.12) | -1.63(-1.92,-1.34) |
| Guatemala | 1770 (1325,2281) | 68.48 (51.4,86.88) | 5565 (4230,7055) | 57.15 (43.6,72.56) | -1.21(-1.54,-0.87) |
| Guinea | 4001 (2682,5441) | 138.72 (94.49,188.75) | 10218 (7010,14354) | 214.15 (148.6,296.88) | 1.51(1.33,1.69) |
| Guinea-Bissau | 760 (527,1056) | 238.14 (166.98,325.04) | 1619 (1119,2219) | 301.79 (210.61,409.12) | 0.96(0.86,1.07) |
| Guyana | 1522 (1197,1873) | 452.09 (356.43,558.52) | 1837 (1329,2398) | 333.29 (243.63,430.45) | -0.43(-0.65,-0.22) |
| Haiti | 7596 (5203,10223) | 310.59 (216.35,410.55) | 14149 (9441,20772) | 262.45 (177.93,377.76) | -0.38(-0.46,-0.3) |
| Honduras | 1739 (1226,2365) | 99.97 (71.29,135.66) | 8764 (6022,11934) | 164.28 (115.07,223.12) | 1.73(1.49,1.97) |
| Hungary | 53310 (40931,65963) | 366.24 (281.39,454.43) | 33381 (25394,42602) | 158.6 (120.24,202.18) | -3.19(-3.42,-2.97) |
| Iceland | 264 (198,335) | 85.94 (64.62,109.12) | 285 (213,363) | 42.74 (32,54.45) | -2.33(-2.47,-2.2) |
| India | 419523 (313125,565863) | 112.99 (84.59,151.7) | 1288789 (978303,1714741) | 119.87 (90.98,158.32) | -0.01(-0.12,0.09) |
| Indonesia | 115988 (83915,153189) | 156.68 (111.99,207.38) | 498832 (338066,681957) | 274.98 (190.41,370.99) | 1.94(1.77,2.12) |
| Iran (Islamic Republic of) | 47696 (36116,61314) | 236.07 (180.06,304.21) | 127943 (99662,159593) | 184.71 (144.19,230.38) | -0.88(-1.01,-0.75) |
| Iraq | 38487 (29592,49376) | 520.45 (401.76,666.38) | 117388 (82975,154877) | 597.85 (429.66,782.31) | 0.01(-0.17,0.18) |
| Ireland | 5283 (4011,6523) | 127.96 (97.23,158.96) | 3293 (2441,4094) | 38.43 (28.63,47.71) | -4.02(-4.24,-3.79) |
| Israel | 4751 (3542,5892) | 98.74 (73.64,122.45) | 6142 (4617,7684) | 45.09 (34.06,56.57) | -3.05(-3.25,-2.85) |
| Italy | 137539 (105911,174222) | 150.53 (116.73,191.06) | 102966 (76168,131308) | 52.61 (39.26,67.21) | -3.7(-3.92,-3.47) |
| Jamaica | 2493 (1915,3171) | 131.96 (101.47,167.94) | 3557 (2433,4739) | 111.18 (76.14,148.23) | -0.34(-0.71,0.02) |
| Japan | 282815 (219856,350546) | 178.2 (137.66,221.48) | 299327 (225670,376596) | 60.97 (46.76,76.87) | -3.81(-3.96,-3.66) |
| Jordan | 4679 (3393,6150) | 438.92 (323.12,571.2) | 16452 (12329,21416) | 278.55 (210.52,362.72) | -1.91(-2.25,-1.57) |
| Kazakhstan | 27572 (20621,35612) | 243.34 (180.73,316.72) | 43869 (32559,56355) | 292.4 (216.13,373.96) | 0.21(-0.26,0.69) |
| Kenya | 4385 (3083,6029) | 69.13 (48.67,95.83) | 15433 (11051,20986) | 91.98 (65.48,125.78) | 1.1(1.02,1.18) |
| Kiribati | 80 (60,104) | 261.31 (194.48,338.4) | 182 (138,238) | 316.07 (241.82,407.63) | 0.63(0.58,0.68) |
| Kuwait | 695 (526,872) | 143.9 (110.26,181.19) | 3300 (2505,4152) | 135.76 (104.45,168.51) | 0.22(-0.75,1.19) |
| Kyrgyzstan | 4723 (3465,6265) | 172.27 (126.13,229.05) | 7005 (5148,9143) | 157.99 (116.69,205.77) | -0.84(-1.24,-0.45) |
| Lao People's Democratic Republic | 4703 (3395,6413) | 290 (210.6,388.08) | 8484 (5914,11487) | 235.14 (164.95,315.51) | -0.89(-0.96,-0.81) |
| Latvia | 8988 (6719,11427) | 249.84 (187.31,318.31) | 10306 (7790,13142) | 225.37 (170.95,287.09) | -0.72(-0.96,-0.47) |
| Lebanon | 4519 (3363,6052) | 242.31 (180.21,323.58) | 8221 (6316,10631) | 128.92 (98.45,167.23) | -2.15(-2.44,-1.86) |
| Lesotho | 627 (437,881) | 90.47 (62.71,127.25) | 1991 (1315,2885) | 231.81 (157.72,327.81) | 4.07(3.53,4.61) |
| Liberia | 1840 (1290,2493) | 186.63 (134.31,250.52) | 3861 (2702,5491) | 233.36 (163,329.19) | 0.74(0.65,0.84) |
| Libya | 2368 (1647,3321) | 142.6 (99.29,201.51) | 9864 (6715,13978) | 213.81 (146.1,300.87) | 1.85(1.61,2.1) |
| Lithuania | 6105 (4574,7787) | 134.94 (100.6,172.19) | 8625 (6492,10947) | 133.07 (100.16,168.75) | 0.12(-0.27,0.51) |
| Luxembourg | 884 (668,1132) | 158.8 (119.99,202.36) | 598 (453,753) | 50 (37.95,62.91) | -3.59(-3.78,-3.41) |
| Madagascar | 4921 (3511,6702) | 127.96 (92.06,171.11) | 11149 (7416,15444) | 150.26 (100.41,204.68) | 0.43(0.35,0.51) |
| Malawi | 2381 (1690,3184) | 89.07 (63.7,118.3) | 7235 (5017,10003) | 130.55 (90.34,178.36) | 1(0.85,1.15) |
| Malaysia | 17371 (13055,21748) | 214.52 (162.5,269.63) | 49592 (38256,61510) | 197.84 (152.83,246.85) | -0.15(-0.27,-0.03) |
| Maldives | 140 (105,180) | 225.04 (170.86,286.53) | 333 (250,428) | 125.27 (94.16,162.56) | -2.3(-2.41,-2.19) |
| Mali | 4619 (3154,6612) | 145.29 (101.91,204.23) | 12531 (8474,17649) | 173.29 (118.93,240.7) | 0.75(0.65,0.86) |
| Malta | 548 (416,686) | 132.77 (100.28,166.52) | 514 (384,646) | 45.6 (34.09,57.26) | -3.65(-3.85,-3.45) |
| Marshall Islands | 47 (35,63) | 367.35 (273.93,482.27) | 91 (65,126) | 369.76 (268.66,495) | -0.02(-0.06,0.01) |
| Mauritania | 1421 (971,1980) | 165.33 (113.44,227.55) | 3225 (2166,4806) | 176.23 (118.49,260.82) | -0.03(-0.24,0.18) |
| Mauritius | 2561 (2005,3158) | 394.84 (308.77,485.01) | 2956 (2265,3727) | 169.47 (130.25,213.99) | -3.92(-4.43,-3.4) |
| Mexico | 53161 (41943,65258) | 147.73 (116.15,182.71) | 85917 (65873,107150) | 72.57 (55.71,90.63) | -2.45(-2.58,-2.33) |
| Micronesia (Federated States of) | 111 (81,150) | 261.22 (190.83,348.45) | 146 (103,200) | 238.99 (172.35,319.82) | -0.4(-0.44,-0.36) |
| Monaco | 136 (95,184) | 160.92 (112.47,219.07) | 107 (78,141) | 89 (65.37,117.05) | -2.07(-2.26,-1.89) |
| Mongolia | 340 (231,460) | 35.51 (24.04,48.55) | 1219 (846,1633) | 61.09 (43.01,82.39) | 1.81(1.55,2.08) |
| Montenegro | 707 (532,928) | 122.78 (92.54,160.87) | 1971 (1436,2664) | 219.66 (160.7,297.12) | 2.21(2.1,2.32) |
| Morocco | 41433 (28741,58282) | 320.12 (223.91,448.61) | 135683 (97362,182374) | 437.07 (315.26,581.58) | 1.22(1.13,1.31) |
| Mozambique | 4845 (3436,6489) | 112.21 (80.04,148.76) | 16451 (10597,22819) | 191.48 (128.73,261.92) | 2.27(2.09,2.46) |
| Myanmar | 54769 (38423,75606) | 291.99 (209.48,396.86) | 102880 (72431,139421) | 248.6 (174.49,336.51) | -0.73(-0.82,-0.63) |
| Namibia | 977 (718,1263) | 206.21 (152.55,266.61) | 2460 (1776,3296) | 230.37 (166.94,307.96) | 0.25(0.02,0.48) |
| Nauru | 13 (9,18) | 375.38 (255.68,495.29) | 19 (13,25) | 389.2 (287.34,517.93) | -0.11(-0.31,0.08) |
| Nepal | 9423 (6299,13519) | 131.39 (89.09,185.42) | 28563 (19361,40044) | 141.83 (96.32,196.16) | 0.15(0.06,0.25) |
| Netherlands | 21061 (15762,26680) | 99.96 (74.96,126.36) | 20436 (15344,25995) | 51.6 (38.54,65.76) | -2.67(-2.93,-2.41) |
| New Zealand | 4481 (3429,5651) | 113.27 (86.23,142.7) | 5621 (4212,7159) | 60.75 (45.42,77.07) | -2.14(-2.32,-1.96) |
| Nicaragua | 996 (746,1274) | 75.95 (57.14,96.68) | 2386 (1734,3155) | 54.84 (40.03,72.2) | -1.05(-1.22,-0.88) |
| Niger | 2583 (1621,3893) | 127.29 (80.58,187.7) | 9867 (6498,14504) | 158.84 (106.32,233.5) | 0.86(0.8,0.91) |
| Nigeria | 52721 (36136,74843) | 145.49 (99.86,203.62) | 105354 (74581,143212) | 150.14 (107,203.19) | -0.02(-0.12,0.07) |
| Niue | 6 (5,8) | 256.53 (195.01,335.6) | 6 (4,7) | 278.52 (212.12,354.85) | 0.07(0.01,0.13) |
| North Macedonia | 9189 (7026,11440) | 570.71 (433.49,709.82) | 18743 (13559,23868) | 696.45 (508.93,882.3) | 0.33(-0.11,0.77) |
| Northern Mariana Islands | 26 (20,35) | 221.7 (171.09,284.51) | 68 (52,86) | 171.32 (132.86,214.34) | -1.25(-1.46,-1.04) |
| Norway | 12452 (9591,15439) | 159.69 (123.1,197.86) | 6152 (4592,7775) | 52.51 (39.22,66.77) | -3.85(-3.94,-3.76) |
| Oman | 1398 (993,1948) | 245.47 (175.01,342.38) | 3701 (2772,4840) | 239.52 (181.96,312.96) | 0.35(0.11,0.59) |
| Pakistan | 68828 (47557,95529) | 137.98 (94.74,191.25) | 197320 (146691,267794) | 196.66 (146.26,267) | 1.05(0.85,1.25) |
| Palau | 27 (21,36) | 322.48 (247.58,425.54) | 60 (45,79) | 332.33 (253.77,430.86) | 0.2(0.15,0.26) |
| Palestine | 2936 (2099,3839) | 396.99 (283.82,519.59) | 5770 (4297,7493) | 298.63 (221.27,384.45) | -0.99(-1.27,-0.72) |
| Panama | 1246 (956,1579) | 91.43 (69.89,115.5) | 3333 (2436,4283) | 73.98 (54.1,94.91) | -0.95(-1.15,-0.75) |
| Papua New Guinea | 1811 (1203,2613) | 144.08 (98.44,204.32) | 5247 (3590,7516) | 148.19 (104.47,209.91) | 0.01(-0.1,0.12) |
| Paraguay | 2508 (1865,3242) | 125.86 (93.66,162.02) | 6630 (4783,8794) | 124.83 (90.18,165.94) | 0.05(-0.09,0.19) |
| Peru | 4604 (3309,6133) | 43.41 (31.28,57.78) | 13034 (9135,17383) | 39.82 (27.84,53.21) | -0.71(-1.1,-0.33) |
| Philippines | 27160 (20334,34517) | 127 (94.3,162.35) | 88977 (66446,113196) | 130.11 (96.79,165.55) | 0.23(0.15,0.31) |
| Poland | 147053 (114523,182411) | 351.56 (275.02,436.08) | 128762 (101004,158465) | 166.37 (130.7,205.25) | -2.8(-2.91,-2.7) |
| Portugal | 53041 (40977,65873) | 398.82 (307.54,495.06) | 27768 (21163,34280) | 88.28 (67.17,108.24) | -5.45(-5.69,-5.22) |
| Puerto Rico | 3524 (2738,4353) | 102.02 (79.21,125.61) | 3970 (2963,4962) | 47.57 (35.58,59.46) | -2.83(-3.03,-2.63) |
| Qatar | 192 (143,245) | 321.35 (238.66,414.85) | 1036 (753,1340) | 199.33 (147.33,259.22) | -1.88(-2.58,-1.18) |
| Republic of Korea | 73086 (54478,93302) | 320.76 (240,409.29) | 87553 (65662,110747) | 93.48 (70.02,118.27) | -4.57(-4.79,-4.35) |
| Republic of Moldova | 5956 (4610,7774) | 160.67 (125.03,209.99) | 10006 (7763,12541) | 163.66 (126.75,205.88) | 0.45(-0.02,0.93) |
| Romania | 81383 (61297,104599) | 334.71 (252.13,428.53) | 120308 (91585,150418) | 289.97 (221.83,361.17) | -0.93(-1.2,-0.67) |
| Russian Federation | 506594 (389416,652579) | 303.73 (233.7,390.13) | 624726 (478224,784130) | 255.33 (195.81,320.55) | -1.27(-1.77,-0.76) |
| Rwanda | 2395 (1670,3416) | 124.52 (89.18,174.74) | 3854 (2515,5462) | 87.11 (57.05,124.7) | -1.92(-2.3,-1.55) |
| Saint Kitts and Nevis | 153 (118,191) | 396.72 (304.66,495.74) | 133 (101,168) | 245.3 (188.97,306.28) | -1.43(-1.62,-1.24) |
| Saint Lucia | 257 (197,322) | 340.03 (263.42,425.92) | 392 (292,498) | 169.83 (126.56,215.66) | -2.9(-3.3,-2.5) |
| Saint Vincent and the Grenadines | 153 (117,189) | 226.11 (174.15,278.74) | 211 (161,262) | 159.49 (121.47,198.42) | -0.96(-1.2,-0.72) |
| Samoa | 147 (107,189) | 207.1 (150.92,263.9) | 289 (216,368) | 228.79 (172.07,290.14) | 0.37(0.28,0.46) |
| San Marino | 39 (28,50) | 102.55 (75.12,132.72) | 46 (32,65) | 48.76 (34.18,68.64) | -2.01(-2.28,-1.73) |
| Sao Tome and Principe | 72 (53,95) | 124.69 (91.46,164.43) | 197 (143,260) | 217.72 (158.58,289.89) | 1.99(1.91,2.08) |
| Saudi Arabia | 14442 (10194,18923) | 299.45 (213.55,390.28) | 45087 (32999,60107) | 283.61 (212.44,379.33) | -0.53(-0.69,-0.38) |
| Senegal | 6951 (5004,9063) | 256.78 (186.52,329.47) | 18255 (13033,25373) | 283.59 (201.39,392.57) | 0.27(0.2,0.34) |
| Serbia | 52509 (40110,66397) | 617.34 (474.3,783.58) | 75059 (55886,97433) | 423.01 (315.99,549.31) | -1.81(-2.07,-1.55) |
| Seychelles | 130 (96,168) | 230.68 (169.8,296.47) | 205 (148,260) | 195.21 (141.69,246.67) | -0.39(-0.56,-0.23) |
| Sierra Leone | 3202 (2292,4337) | 175.69 (127.07,237.29) | 6975 (4698,9624) | 221.48 (151.59,302.92) | 1.02(0.85,1.19) |
| Singapore | 4757 (3685,5831) | 248.23 (192.46,302.72) | 3685 (2758,4703) | 43.95 (32.96,56.11) | -5.72(-5.94,-5.49) |
| Slovakia | 16652 (12601,21404) | 280.85 (213.32,359.46) | 18542 (13736,23790) | 188.85 (139.28,242.52) | -1.43(-1.56,-1.29) |
| Slovenia | 5961 (4582,7414) | 241.25 (187.52,298.79) | 4695 (3555,5839) | 89.44 (67.51,111.49) | -3.26(-3.43,-3.09) |
| Solomon Islands | 160 (109,221) | 162.88 (113.51,224.77) | 455 (311,648) | 173.45 (118.84,246.36) | 0.1(0.06,0.15) |
| Somalia | 1360 (834,2035) | 91.31 (57.22,134.02) | 3834 (2350,5866) | 96.89 (60.12,147.16) | 0.23(0.18,0.29) |
| South Africa | 19351 (13899,25181) | 109.27 (78.95,141.83) | 67970 (52354,85592) | 175.16 (135.22,220.65) | 1.8(1.3,2.3) |
| South Sudan | 1897 (1260,2752) | 92.25 (61.39,131.48) | 2751 (1828,4003) | 101.39 (68.16,146.95) | 0.15(-0.02,0.33) |
| Spain | 96163 (72931,119787) | 173.6 (131.19,216.23) | 61902 (46627,78176) | 50.51 (38.04,63.74) | -4.04(-4.21,-3.86) |
| Sri Lanka | 18540 (13936,23431) | 229.76 (173.05,291.93) | 50183 (33568,66993) | 208.42 (140.05,278.49) | 0.31(0.02,0.61) |
| Sudan | 22660 (14939,32493) | 279.58 (185.54,396.35) | 49784 (32292,70108) | 297.55 (196.25,412.79) | 0.06(0,0.12) |
| Suriname | 417 (306,526) | 184.44 (135.64,231.94) | 954 (669,1258) | 159.68 (111.64,210.92) | -0.42(-0.69,-0.15) |
| Sweden | 17071 (12998,21707) | 98.24 (74.75,125.09) | 12840 (9625,16422) | 48.72 (36.5,62.06) | -2.48(-2.69,-2.26) |
| Switzerland | 11748 (8857,14965) | 99.66 (74.92,127.01) | 8387 (6225,10645) | 37.33 (28,47.57) | -3.13(-3.26,-3) |
| Syrian Arab Republic | 10447 (7443,13989) | 234.14 (168.13,315.29) | 27733 (19799,38334) | 249.91 (180.9,342.13) | -0.31(-0.54,-0.09) |
| Taiwan (Province of China) | 24180 (18591,30093) | 182.9 (139.74,227.56) | 31510 (23321,39747) | 72.43 (53.29,91.52) | -3.06(-3.23,-2.89) |
| Tajikistan | 4502 (3157,5974) | 179.44 (125.81,237.87) | 9795 (7238,12919) | 215.56 (158.91,283.84) | 0.41(0.04,0.79) |
| Thailand | 35586 (26180,45641) | 123.38 (90.77,158.22) | 104524 (76356,138983) | 96.14 (70.13,128.1) | -1.37(-1.57,-1.16) |
| Timor-Leste | 192 (138,257) | 109.15 (77.64,146.45) | 1266 (797,1778) | 173.64 (111.61,244.23) | 1.51(1.44,1.58) |
| Togo | 1436 (1003,1913) | 153.91 (109.31,206.06) | 5691 (3844,8095) | 199.88 (137,279.41) | 0.75(0.59,0.9) |
| Tokelau | 4 (3,5) | 301.25 (225.43,387.35) | 4 (3,5) | 269.81 (198.71,342.47) | -0.42(-0.45,-0.38) |
| Tonga | 61 (45,79) | 127.41 (95.76,162.68) | 108 (80,141) | 140.81 (104.47,184.17) | 0.39(0.29,0.49) |
| Trinidad and Tobago | 1975 (1523,2454) | 265.2 (205.63,328.39) | 2907 (2083,3860) | 153.7 (110.75,204.42) | -2.04(-2.27,-1.8) |
| Tunisia | 8312 (6044,11377) | 201.25 (146.67,272.12) | 25794 (17120,35365) | 210.01 (138.42,285.26) | -0.09(-0.24,0.06) |
| Turkmenistan | 2537 (1858,3346) | 145.41 (106.61,191.7) | 10700 (7571,14987) | 291.89 (209.91,405.44) | 2.09(1.66,2.52) |
| Tuvalu | 11 (8,15) | 213.69 (155.46,274.36) | 17 (12,22) | 185.45 (134.74,247.97) | -0.55(-0.61,-0.5) |
| Turkey | 58865 (43496,75883) | 204.77 (151.93,263.07) | 143809 (106685,185134) | 168.11 (123.81,217.39) | -0.54(-0.83,-0.25) |
| Uganda | 4675 (3238,6584) | 96.58 (66.48,134.18) | 10494 (7333,14538) | 95.3 (67.3,131.71) | -0.57(-0.81,-0.33) |
| Ukraine | 184521 (140709,238850) | 267.3 (206.45,348.07) | 156746 (112322,212428) | 194.07 (138.39,262.15) | -1.63(-1.91,-1.35) |
| United Arab Emirates | 1117 (795,1510) | 350.39 (251.07,467.09) | 6487 (4803,8306) | 319.85 (239.26,402.77) | 1.45(0.88,2.03) |
| United Kingdom | 106968 (82285,136830) | 107.53 (82.58,137.69) | 61951 (46607,78416) | 40.71 (30.52,51.48) | -3.41(-3.63,-3.2) |
| United Republic of Tanzania | 5014 (3487,7072) | 64.48 (45.32,91.29) | 23276 (16017,32600) | 117.16 (80.9,162.91) | 1.9(1.73,2.07) |
| United States of America | 309137 (237570,388980) | 91.24 (70.01,114.66) | 539400 (412902,662527) | 86.66 (66.94,106.19) | -0.6(-0.84,-0.36) |
| United States Virgin Islands | 91 (67,115) | 136.16 (102.1,172.82) | 148 (110,197) | 80.18 (59.24,106.7) | -1.56(-1.69,-1.42) |
| Uruguay | 5516 (4244,7011) | 137.97 (105.4,175.55) | 6073 (4602,7682) | 95.84 (73.08,120.99) | -1.35(-1.53,-1.16) |
| Uzbekistan | 11976 (8724,15593) | 109.22 (79.83,141.79) | 44413 (32566,57674) | 195.54 (144.59,254.13) | 1.51(1.2,1.83) |
| Vanuatu | 113 (81,152) | 239.93 (173.33,319.1) | 327 (236,431) | 235.73 (170.94,311.17) | -0.22(-0.28,-0.15) |
| Venezuela (Bolivarian Republic of) | 6888 (5250,8767) | 82.33 (62.86,104.66) | 23140 (16488,31232) | 84.61 (60.25,114.51) | -0.26(-0.5,-0.01) |
| Viet Nam | 63853 (46312,86024) | 182.62 (131.83,243.39) | 195048 (133490,258885) | 234.84 (161.26,309.41) | 1.07(0.9,1.23) |
| Yemen | 10498 (6943,15179) | 266.84 (177.24,378.32) | 38247 (24752,56586) | 333.09 (219.43,485.73) | 0.65(0.61,0.69) |
| Zambia | 2764 (1912,3878) | 135.26 (94.3,188.56) | 9540 (6851,13561) | 192.34 (137.94,267.22) | 1.06(0.96,1.15) |
| Zimbabwe | 3570 (2663,4630) | 120.54 (89.97,155.01) | 10036 (7212,13413) | 196.2 (142.83,260.77) | 2.18(1.7,2.66) |
