# Supplementary material for: The persistent challenge of ischemic stroke burden from high fasting plasma glucose: a global perspective
Source: Front Endocrinol (Lausanne). 2025 May 6;16:1490428. doi: 10.3389/fendo.2025.1490428 (PMC12088946; doi:10.3389/fendo.2025.1490428)
Supplement: Supplementary file 5 [file Table3.docx]

Table S3 Age-standardised DALY rate and mortality, and percentage change from 1990 globally and by SDI quintile

| Location | DALYs changes due to population-level determinants* | | | Mortality changes due to population-level determinants* | | |
| --- | --- | --- | --- | --- | --- | --- |
|  | Ageing | Population | EC | Ageing | Population | EC |
| Global |  | | | | | |
| Both | 1993415.87 (35.19%) | 5057416.86 (89.29%) | -1386523.90 (-24.48%) | 140060.37 (42.67%) | 307570.77 (93.7%) | -119375.79 (-36.37%) |
| Male | 1207751.63 (38.66%) | 2512747.29 (80.44%) | -596703.95 (-19.1%) | 79975.32 (48.74%) | 138220.97 (84.24%) | -54112.62 (-32.98%) |
| Female | 802141.78 (32.13%) | 2530833.44 (101.37%) | -836354.14 (-33.5%) | 59358.14 (37.48%) | 167674.99 (105.87%) | -68658.24 (-43.35%) |
| High SDI |  | | | | | |
| Both | 550862.57 (114.22%) | 1171928.17 (243%) | -1240515.06 (-257.22%) | 51336.15 (102.35%) | 95890.45 (191.19%) | -97071.34 (-193.54%) |
| Male | 329206.51 (207.11%) | 663178.10 (417.21%) | -833429.42 (-524.32%) | 30040.88 (233.9%) | 49294.88 (383.81%) | -66492.21 (-517.71%) |
| Female | 225972.86 (104.19%) | 525101.16 (242.1%) | -534179.51 (-246.29%) | 21037.48 (83.15%) | 46315.82 (183.07%) | -42054.12 (-166.23%) |
| High-middle SDI |  | | | | | |
| Both | 2432895.46 (72.9%) | 1773642.94 (107.24%) | -1949798.57 (-80.14%) | 123070.76 (82.67%) | 215527.3 (144.77%) | -189723.57 (-127.44%) |
| Male | 1307922.67 (71.92%) | 940674.32 (98.3%) | -918409.34 (-70.22%) | 65756.86 (87.04%) | 104259.55 (138.01%) | -94472.68 (-125.06%) |
| Female | 1060287.15 (78.7%) | 834435.90 (125.01%) | -1099563.58 (-103.7%) | 55789.69 (85.91%) | 110142.49 (169.62%) | -100996.12 (-155.53%) |
| Middle SDI |  | | | | | |
| Both | 910235.03 (39.11%) | 1595706.99 (68.56%) | -178439.28 (-7.67%) | 72659.96 (39.85%) | 132668.12 (72.75%) | -22976.65 (-12.6%) |
| Male | 498838.83 (39.28%) | 814468.77 (64.14%) | -43386.30 (-3.42%) | 39640.90 (42.74%) | 65016.51 (70.1%) | -11915.53 (-12.85%) |
| Female | 411494.98 (39.36%) | 776282.98 (74.25%) | -142266.48 (-13.61%) | 32535.30 (37.44%) | 67359.79 (77.51%) | -12988.47 (-14.95%) |
| Low-middle SDI |  | | | | | |
| Both | 222533.11 (16.25%) | 929603.44 (67.87%) | 217497.74 (15.88%) | 19738.12 (24.33%) | 91221.65 (112.45%) | -29840.59 (-36.79%) |
| Male | 91656.81 (12.47%) | 477164.16 (64.93%) | 166024.35 (22.59%) | 8462.86 (18.51%) | 46346.91 (101.35%) | -9080.14 (-19.86%) |
| Female | 127035.47 (19.79%) | 452690.06 (70.51%) | 62307.34 (9.7%) | 11141.18 (29.79%) | 45242.65 (120.99%) | -18991.02 (-50.79%) |
| Low SDI |  | | | | | |
| Both | 1977.83 (0.12%) | 1440101.99 (83.86%) | 275150.58 (16.02%) | -351.78 (-1.94%) | 16312.13 (90%) | 2164.06 (11.94%) |
| Male | 18680.21 (1.98%) | 784042.50 (83.2%) | 139583.80 (14.81%) | -507.30 (-5.28%) | 8726.96 (90.79%) | 1392.57 (14.49%) |
| Female | -13328.08 (-1.64%) | 689279.43 (84.91%) | 135832.95 (16.73%) | 106.97 (-1.24%) | 7706.23 (89.24%) | 822.46 (9.52%) |

DALY–disability-adjusted life years, EC–Epidemiological change, SDI–sociodemographic index,*Percentages contribute to the total changes.
